# Progress on Optimizing Miscanthus Biomass Production for the European Bioeconomy: Results of the EU FP7 Project OPTIMISC

**DOI:** 10.3389/fpls.2016.01620

**Published:** 2016-11-18

**Authors:** Iris Lewandowski, John Clifton-Brown, Luisa M. Trindade, Gerard C. van der Linden, Kai-Uwe Schwarz, Karl Müller-Sämann, Alexander Anisimov, C.-L. Chen, Oene Dolstra, Iain S. Donnison, Kerrie Farrar, Simon Fonteyne, Graham Harding, Astley Hastings, Laurie M. Huxley, Yasir Iqbal, Nikolay Khokhlov, Andreas Kiesel, Peter Lootens, Heike Meyer, Michal Mos, Hilde Muylle, Chris Nunn, Mensure Özgüven, Isabel Roldán-Ruiz, Heinrich Schüle, Ivan Tarakanov, Tim van der Weijde, Moritz Wagner, Qingguo Xi, Olena Kalinina

**Affiliations:** ^1^Department of Biobased Products and Energy Crops, Institute of Crop Science, University of HohenheimStuttgart, Germany; ^2^Institute of Biological, Environmental and Rural Sciences, Aberystwyth UniversityAberystwyth, UK; ^3^Department of Plant Breeding, Wageningen UniversityWageningen, Netherlands; ^4^Dienst Landbouwkundig Onderzoek, Wageningen UR Plant BreedingWageningen, Netherlands; ^5^Julius Kühn-InstitutBraunschweig, Germany; ^6^ANNA - The Agency for Sustainable Management of Agricultural LandscapeFreiburg, Germany; ^7^Department of Plant Physiology, Russian State Agrarian University–Moscow Timiryazev Agricultural AcademyMoscow, Russia; ^8^Plant Sciences Unit, Institute for Agricultural and Fisheries ResearchMelle, Belgium; ^9^Blankney EstatesBlankney, UK; ^10^The Institute of Biological and Environmental Sciences, University of AberdeenAberdeen, UK; ^11^Faculty of Agriculture and Natural Sciences, Konya Food and Agriculture UniversityKonya, Turkey; ^12^German Agrarian CentrePotash, Ukraine; ^13^Dongying Agricultural InstituteDongying, China

**Keywords:** Miscanthus, genotypes, stress tolerance, marginal land, value chains, costs, LCA, bioeconomy

## Abstract

This paper describes the complete findings of the EU-funded research project OPTIMISC, which investigated methods to optimize the production and use of miscanthus biomass. Miscanthus bioenergy and bioproduct chains were investigated by trialing 15 diverse germplasm types in a range of climatic and soil environments across central Europe, Ukraine, Russia, and China. The abiotic stress tolerances of a wider panel of 100 germplasm types to drought, salinity, and low temperatures were measured in the laboratory and a field trial in Belgium. A small selection of germplasm types was evaluated for performance in grasslands on marginal sites in Germany and the UK. The growth traits underlying biomass yield and quality were measured to improve regional estimates of feedstock availability. Several potential high-value bioproducts were identified. The combined results provide recommendations to policymakers, growers and industry. The major technical advances in miscanthus production achieved by OPTIMISC include: (1) demonstration that novel hybrids can out-yield the standard commercially grown genotype *Miscanthus x giganteus;* (2) characterization of the interactions of physiological growth responses with environmental variation within and between sites; (3) quantification of biomass-quality-relevant traits; (4) abiotic stress tolerances of miscanthus genotypes; (5) selections suitable for production on marginal land; (6) field establishment methods for seeds using plugs; (7) evaluation of harvesting methods; and (8) quantification of energy used in densification (pellet) technologies with a range of hybrids with differences in stem wall properties. End-user needs were addressed by demonstrating the potential of optimizing miscanthus biomass composition for the production of ethanol and biogas as well as for combustion. The costs and life-cycle assessment of seven miscanthus-based value chains, including small- and large-scale heat and power, ethanol, biogas, and insulation material production, revealed GHG-emission- and fossil-energy-saving potentials of up to 30.6 t CO_2eq_ C ha^−1^y^−1^ and 429 GJ ha^−1^y^−1^, respectively. Transport distance was identified as an important cost factor. Negative carbon mitigation costs of –78€ t^−1^ CO_2eq_ C were recorded for local biomass use. The OPTIMISC results demonstrate the potential of miscanthus as a crop for marginal sites and provide information and technologies for the commercial implementation of miscanthus-based value chains.

## Introduction

Miscanthus is a C4 perennial rhizomatous grass native to East Asia. The genus *Miscanthus* has its origins in the tropics and subtropics, but its various species are found over a wide climatic range throughout East Asia (Greef and Deuter, 1993). The remarkable ability of miscanthus to adapt to different environments (Numata, 1974) makes this novel crop suitable for production over a range of European and North American climatic conditions. Miscanthus was first cultivated in Europe in the 1930s, when it was introduced from Japan. Today it has become a leading candidate crop for production of lignocellulosic feedstocks for both bioenergy and material uses, thanks to its rapid biomass accumulation in temperate climates (Clifton-Brown et al., 2017).

Field experiments with the only genotype currently commercially available, *Miscanthus x giganteus*, a clone-based interspecies hybrid, have revealed its great photosynthetic efficiency, high biomass yield capacity, low input demands and good tolerance of temperate climates, and many of the characteristics that make miscanthus an ideal biomass crop (Lewandowski et al., 2000; Dohleman and Long, 2009; Heaton et al., 2010; van der Weijde et al., 2013; Davey et al., 2016). Analyses of the environmental impacts of miscanthus cultivation on a range of factors, including greenhouse gas mitigation, show that the benefits outweigh the costs in most cases (McCalmont et al., 2015). At present, only about 20,000 ha of miscanthus are commercially grown in the EU, mostly in the UK (10,000 ha), France (4000 ha), Germany (4000 ha), Switzerland (500 ha), and Poland (500 ha). There are several reasons for the low implementation and even decreasing cultivation area of miscanthus in Europe (Lewandowski, 2016).

Biomass production costs for miscanthus are presently too high to compete commercially with fossil fuels on an energy basis. The high biomass production costs for miscanthus result from insufficient development of agricultural production technology, accompanied by additional costs for agricultural inputs, land and labor for a relatively low-value biomass. Although they are amortized over a production period of 10–25 years, initial establishment costs for miscanthus are still comparatively high. This is because the only commercially available genotype *Miscanthus* × *giganteus* is a triploid hybrid that does not produce viable seeds. Consequently, costly establishment via rhizome or *in vitro* propagation has to be performed (Xue et al., 2015). Miscanthus is also new to farmers and they have neither the knowledge nor the technical equipment to cultivate it. Thus, inefficient production technology is currently limiting its widespread uptake as a biomass crop.

There are no stable markets for miscanthus biomass and relevant applications are low-value. Farmers are hesitant to cultivate miscanthus because it involves dedicating their fields to long-term biomass production. They will only be willing to do this once biomass markets are stable or if long-term contracts are available (Wilson et al., 2014). The main use of lignocellulosic biomass from perennial crops is as a solid fuel for heat and power generation—a comparatively low-value use, its profitability being ultimately determined by the price of fossil fuels. In Europe, subsidies are generally necessary for bioenergy products to be able to compete in retail energy markets—with the notable exception of forest wood and forestry by-products that cannot be used for wood material products. Therefore, also higher-value applications for miscanthus biomass are required in order to provide attractive market options.

There are no miscanthus varieties adapted to different site characteristics and biomass use options. In Europe, *Miscanthus* × *giganteus* is the only genotype commercially available. Major barriers to the breeding of miscanthus varieties are the high costs involved and the long breeding periods, necessary because most yield- and quality-relevant parameters are not quantifiable until after the establishment phase of 2–3 years.

The EU project OPTIMISC (Optimizing Miscanthus Biomass Production) was initiated in 2012 with the objective of providing solutions to remove some of these barriers to miscanthus production. More specifically, the following research goals were the starting point for the OPTIMISC R&D activities (see also **Table 5**).

– Identification of novel miscanthus genotypes adapted to different climatic conditions and to adverse and marginal site conditions, such as cold, drought, and salinity;
– Improvement of productivity and yield stability of miscanthus;
– Reduction of biomass production and supply costs by demonstrating large-scale field production based on seeded hybrids and by optimizing harvesting regime and logistics;
– Improving marketing opportunities for miscanthus biomass by assessing genetic determinants of biomass quality, identifying novel value chains and developing logistic technology;
– Optimization of miscanthus-based product supply chains in terms of costs and environmental performance.

To address these objectives, miscanthus bioenergy and bioproduct chains were optimized by trialing diverse germplasm types over a range of sites across central Europe, Ukraine, Russia, and China. The key traits that currently limit the potential of miscanthus were analyzed, high-value bioproducts identified and the combined results modeled to provide recommendations to policy makers, growers, and industry.

Here we provide a summary of the OPTIMISC project's achievements and discuss their relevance for the advancement of miscanthus development and its implementation.

## Materials and methods

**Figure 1:**
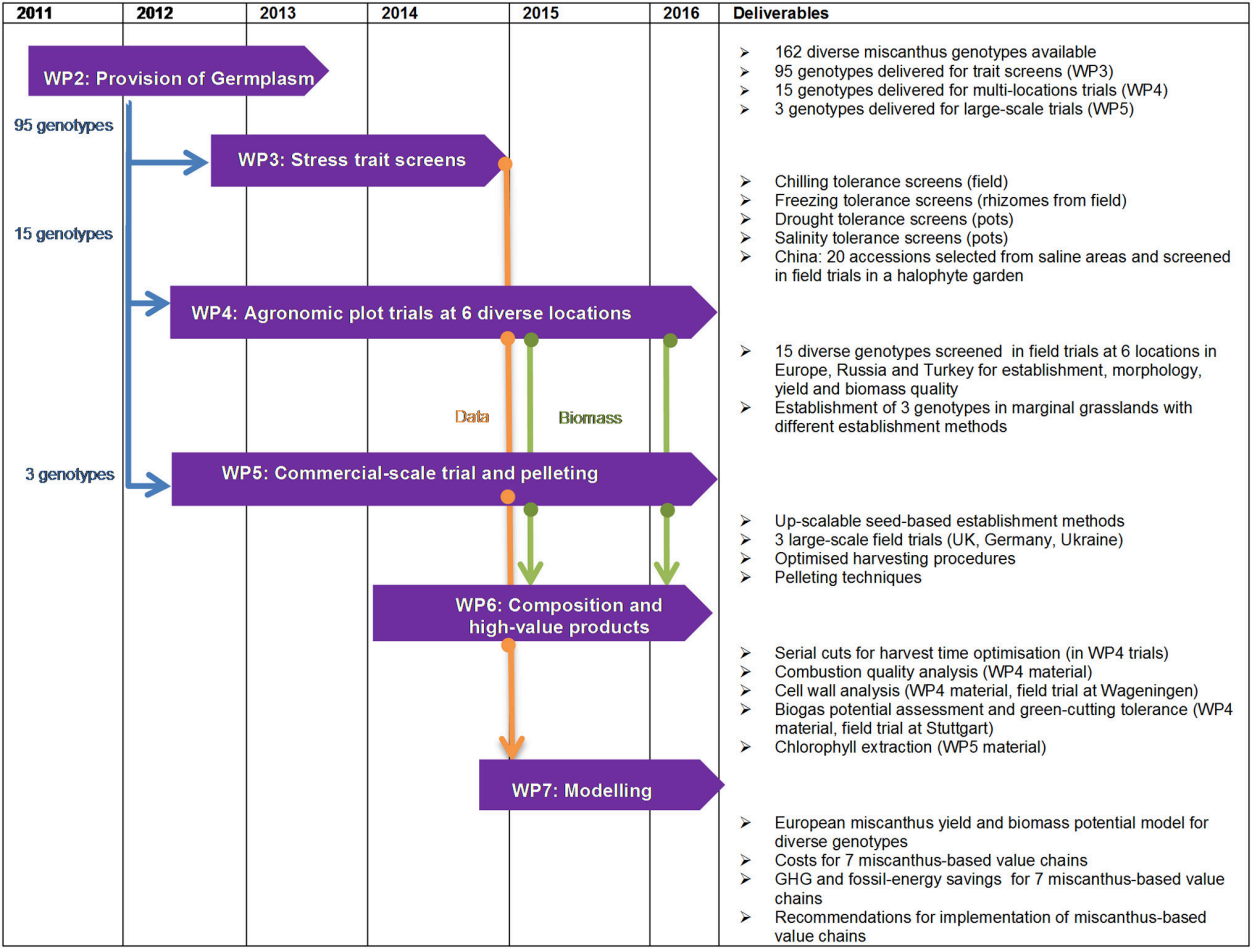
**Overview of the research and development activities in OPTIMISC work packages (WP)**.

Figure 1 gives an overview of the organization of the research and development activities of the OPTIMISC project in different work packages (WP). The overall project co-ordination was performed in WP1. Diverse miscanthus germplasm (provided by IBERS from Aberystwyth University, the Department of Plant Breeding from Wageningen University (WU), ILVO, Schwarz and the Dongying Agricultural Institute) was propagated (Work package 2; WP2) and experiments were conducted on different scales in laboratories, glasshouses, field plots and in pre-commercial scale field trials. About 100 genotypes were studied under controlled conditions to obtain insights into the available genetic variation in the miscanthus genepool for traits such as growth under low water input, saline conditions and low temperatures (WP3). Fifteen genotypes were screened on field sites in the UK, Germany, the Netherlands, Turkey, Ukraine, Russia, and China (WP4). Harvest systems designed to optimize biomass quality and costs were applied on large-scale farm demonstration trials with one to three genotypes (WP5). The composition of the biomass was investigated with regard to its quality for various energy supply chains and material uses (WP6). Yields and miscanthus-based value chains were modeled with the objective of identifying the best options for different climatic settings and biomass uses (WP7). The following sections give a more detailed description of the methods of the experimental work packages.

### Work package 2: provision of germplasm and plant material

The objective of WP2 was to provide novel miscanthus germplasm to be screened in laboratory, glasshouse and field trials in WPs3, 4, and 5 (Figure 1).

Miscanthus germplasm was provided by several partners from the UK, the Netherlands, Germany, Belgium and China. Table 1 summarizes the germplasm used by species and provider.

**Table 1:** **Miscanthus germplasm investigated in OPTIMISC and its origin**.

**Germplasm**	**Total**	Provider^*^	**Number of genotypes**
*Miscanthus* × *giganteus*	1	Schwarz	1
*Miscanthus sinensis*	111	IBERS	6
		WU	100
		Schwarz	4
		ILVO	1
*Miscanthus sacchariflorus*	35	IBERS	15
		Dongying	20
*Miscanthus* hybrids (novel breeds)	16	IBERS	16
Total number of genotypes investigated	163		

* *IBERS, Aberythwyth University (UK); WU, Department of Plant Breeding, Wageningen University (the Netherlands); Schwarz, Schwarz, Braunschweig (Germany), ILVO, Institute for Agricultural and Fisheries Research (Belgium); Dongying, Dongying Agricultural Institute (China)*.

By 2013, about 95 miscanthus genotypes had been successfully transferred to *in vitro* culture, mostly from rhizome buds. However, several tests had to be performed to find the appropriate source material (rhizome pieces, stem segments, immature inflorescences, and seeds) and media compositions for all genotypes. The standard medium used for the storage of the plants was made by stirring a ready-mixed basic MS-medium, saccharose and the phytohormone BAP in distilled water. Clones were supplied as *in vitro* cultures by partner Schwarz to WP3-participating partners who then propagated them further *in vitro* to use in the trait screens in controlled environments in WP3.

A subset of 15 germplasm types (11 genotypes by clones, and 4 seed populations—a total of 22,200 plants) were produced for the WP4 multi-location trials. The clone-based genotypes were transferred from *in vitro* vessels to soil in multi-trays. These were covered with film for approximately 10 days to increase air humidity and to keep leaf transpiration low. High temperature (25°C) in the glasshouse in that period was also advantageous for root growth. The multi-trays were kept in the glasshouse for 3 weeks before the plants were sent to the WP4 partner locations. For the propagation of seed-based populations, seeds were sown into multi-trays and kept for 6 weeks in the glasshouse at 25°C before being sent to the WP4 partners.

In WP5, seed-based hybrids were sown in plugs under glasshouse conditions and planted at the sites in Blankney (UK) in 2012, and in Stuttgart (Germany) and Potash (Ukraine) in 2013. They were produced through a close collaboration between the OPTIMISC commercial partner Blankney Estates and Bell's nurseries in Lincoln, UK. Bell used vacuum sowing for the modules, which were then raised for 6 weeks in the glasshouse at 25°C. The seeds for plug plantings in 2013 were produced in German breeding nursery trials. Plug plants with 2–5 stems and about 20–30 cm height were transplanted using hand and mechanical planting systems.

### Work package 3: stress trait screen in controlled environments and in the field

The objective of WP3 was to identify miscanthus genotypes tolerant to abiotic stresses by performing screening for cold, drought, and salinity tolerance. Combining resilience traits through breeding is expected to result in future hybrids better suited to marginal conditions than the standard genotype *Miscanthus* × *giganteus* (*M*. × *giganteus*) (Fonteyne et al., 2016c). Trait screens were performed in controlled environments on chilling tolerance by the partner ILVO in Belgium, on drought by the partner IBERS in the UK, and on salinity by the partner DLO in the Netherlands. Chilling tolerance was screened in field trials and in a saline field by the partner ILVO in Belgium, and salinity tolerance in a saline environment in China by the partner Dongying.

#### Quantifying variation in low-temperature tolerance of (i) the overwintering rhizome (ii) spring growth

Ninety-five of 162 genotypes were successfully *in vitro* cloned for use in the abiotic stress experiments. Chilling and winter frost tolerance were tested by planting clones in the field (Table 2). In the first winter after planting, rhizomes were dug out and cleaned, cut into 10-cm lengths with at least one viable bud, and exposed to different freezing temperatures in a temperature-controlled bath. The rhizomes were left to thaw and then allowed to grow in optimal conditions. Frost tolerance was quantified by determining the temperature at which 50% of the rhizomes of each genotype were killed (LT_50_). A total of 95 genotypes were tested for this trait. Shoot frost tolerance and winter survival were evaluated in field trials. Chilling tolerance (102 genotypes) was investigated by studying early vigor at the beginning of the growing season in field trials and by measuring growth under chilling stress in growth chambers. From these experiments, a number of growth traits were calculated (including longest shoot, no. of leaves and shoots, growth rate, leaf formation rate) and these traits were analyzed to determine which can best be used to describe early vigor and chilling tolerance, and which are most reproducible and useful to breeders.

**Table 2:** **Overview of trait screen experiments**.

**Trait**	**No of genotypes screened**	**Method's**	Location^*^	**References**
Chilling	56	Growth chambers	ILVO	Fonteyne et al., 2016a
Chilling—early vigor	102	Field trial (chilling tolerance trial)	ILVO, WP4 partners	
Frost—winter survival—shoot frost tolerance	102	Field trial (chilling tolerance trial, mini-plots trial, multi-location trial)	ILVO, WP4 partners	Fonteyne et al., 2016b
Frost—rhizomes	95	Rhizomes pieces temperature-controlled bath	ILVO	Fonteyne et al., 2016b
Drought	87	Greenhouse, pots and 1-m long plastic tubes	IBERS	van der Weijde et al., 2016b
Salinity	70	Greenhouse, hydroponics system	DLO	

* *IBERS, University of Aberythwyth (UK); DLO, Wageningen University and Research Centre, Plant Research International (the Netherlands); ILVO, Institute for Agricultural and Fisheries Research (Belgium)*.

#### Screens on drought and salinity tolerance under controlled conditions

For the preferred protocol for screening for drought tolerance, *in vitro*-grown plants were transferred and established in soil conditions (Table 2). After 1 year of establishment in soil, the senesced year-1 biomass was harvested in the spring. Like-sized, newly emerged tillers were subsequently selected per genotype (*n* = 20) once emerged, and grown in 5-inch pots (37 genotypes) and 1-m long pipes (50 genotypes), where water was withheld for 12 and 28 days, respectively. Half the plants were harvested at the end of the drought treatment. For the other half watering was resumed for a period of recovery. Growth measurements included leaf elongation of the newest emerging leaf, number of tillers, and fresh and dry weight of leaves, stems, and roots at the time of harvest. A subsequent similar evaluation of six selected genotypes exhibiting a variety of responses to drought stress included additional physiological traits: Stomatal conductance, stomata count, and maximum efficiency of photosystem II (Fv/Fm).

For evaluation of salt tolerance, 70 *in vitro*-grown genotypes were transferred to a hydroponics system in the greenhouse and grown under normal conditions as well as saline conditions (150 mM NaCl added to the growth medium) (Table 2). The salt treatment was continued for 3 weeks. During the stress period, tiller number, leaf elongation and leaf elongation rate, plant height, and chlorophyll content were measured, and senescence was visually assessed. At the end of the stress period, the plants were harvested and shoot and root fresh and dry weight determined. The dried samples were used for determination of ion contents (Na^+^, K^+^, Ca^2+^, Cl^−^, Mg^2+^, ${\text{SO}}_{4}^{2-}$, ${\text{PO}}_{4}^{3-}$). A selected set of genotypes was further evaluated in pots. These included *in vitro*- and hydroponics-propagated plants, as well as plants started from rhizomes (collected in the field). Rhizomes were cut into pieces of similar size. The plants were subjected to normal conditions (no added salt), 150 and 250 mM NaCl salinity after 3 weeks of acclimation. The salt treatment was continued for 6 weeks, after which the plants were harvested. Leaves, stems, and root fresh and dry weights were determined at harvest, and the dry material was used for determination of ion contents (Na^+^, K^+^, Ca^2+^, Cl^−^, Mg^2+^, ${\text{SO}}_{4}^{2-}$, ${\text{PO}}_{4}^{3-}$).

#### Field trials in halophyte gardens in China

Twenty novel natural miscanthus germplasm types, selected under saline conditions in China and grown in pots from seeds, were planted in the field at Dongying in China in 2013. The field trials were designed as fully randomized blocks with three replicates. There were two trials at different sites, one with almost normal soil and the other with saline soil. The salinity levels, measured by electrical conductivity (EC), were 1–2 S/m at site A, and 2–8 S/m at site B. Growth parameters were measured throughout 2014.

### Work package 4: multi-location agronomic plot trials

The objectives of WP4 were to screen novel miscanthus germplasm under field conditions in diverse European climates, and to establish miscanthus into marginal grasslands. Assessments were performed on the productivity and yield stability of these genotypes and to identify those that yield higher than the standard genotype *M*. × *giganteus*. Further, biomass samples for quality analysis were delivered to WP6 and data for yield modeling and cost and life cycle assessment to WP7.

#### Agronomic plot trials at 6 locations in Europe, Turkey, and Russia

In 2012, 15 miscanthus types (see Table 4 for description) were provided through WP2 for plot trials in Turkey near Adana, in Germany near Stuttgart, in Ukraine near Potash, in the Netherlands at Wageningen, in the United Kingdom near Aberystwyth, and in Russia near Moscow. At each site, three replicate 25 m^2^ plots were planted with 49 plants (plugs) per plot (resulting in a density close to 2 plants per m^2^) in randomized blocks. For the remainder of this paper, the sites are referred to by the name of the nearest town. The six trial sites cover a wide range of climate and soil conditions (Table 3). The field trials were established mostly on arable or horticultural land except in Aberystwyth, where the trial was set up on marginal (low-quality) grassland.

**Table 3:** **Location characteristics and previous land use of the six OPTIMISC field trials established in May 2012**.

**Country**	**Location name**	**Latitude**	**Longitude**	**Altitude (m)**	**Previous land use**	**Annual air temperature,°C**	**Annual rainfall, mm**
Turkey	Adana	37.00	35.00	27	Arable	19.0	575.2
Germany	Stuttgart	48.74	8.93	463	Arable	9.8	725.4
Ukraine	Potash	48.89	30.44	237	Arable	8.9	537.2
Netherlands	Wageningen	51.59	5.39	10	Horticultural	10.3	826.4
UK	Aberystwyth	52.43	–4.01	39	Grassland	9.7	1038.1
Russia	Moscow	55.50	37.33	140	Arable	4.1	644.0

**Table 4:** **Miscanthus genotypes used in plot-based field trials**.

**Genotype ID**	**Provider**	**Species**	**Propagation Method**
OPM-01	IBERS	*Miscanthus sacchariflorus*	*In vitro tillering to produce plug plants*
OPM-02	IBERS		
OPM-03	IBERS		
OPM-04	IBERS		
OPM-05	IBERS	*M. sinensis × M. sacchariflorus* hybrids,	*In vitro tillering to produce plug plants*
OPM-06	IBERS		
OPM-07	IBERS		
OPM-08	IBERS		
OPM-09	IBERS	*Miscanthus* × *giganteus*	
OPM-10	Schwarz	*M. sinensis × M. sacchariflorus* hybrids	
OPM-11	IBERS	*Miscanthus sinensis*.	*In vitro*
OPM-12	IBERS		Seedlings raised in plugs
OPM-13	WUR		
OPM-14	WUR		
OPM-15	IBERS		

Before planting out the plugs, the ground was prepared as follows: Weeds were removed with glyphosate or by mechanical methods, inversion plowed and harrowed or rotivated to produce a fine tilth. The plugs were planted between 15 and 25 May 2012 at all sites and watered to provide a good hydraulic contact between the soil and plug. Post-planting herbicide was not applied in the first year, and weeds were controlled mechanically.

In the first year (2012), fertilizer was applied at all the sites at rates of 44 and 110 kg ha^−1^ phosphorus (P) and potassium (K), respectively. No nitrogen (N) fertilizer was applied that year to avoid stimulating weed growth. In the following year, fertilizer was applied at a rate of 100 kg ha^−1^ P, 140 kg ha^−1^ K and 60 kg ha^−1^ N to ensure non-limiting crop nutrition at all sites.

A drip irrigation system was installed in Turkey. Irrigation amounts in years 1 and 2 (2012 and 2013) were 75% of evapotranspiration (ETp). In years 3 and 4, irrigation levels were lowered to 25% of ETp, to help identify the most drought-tolerant germplasm.

Yields were estimated in the spring following the growing season by harvesting nine plants in the middle of the plots (4.6 m^2^) at a cutting height of 5 cm above the soil surface. Subsamples were weighed and oven-dried to calculate yield as tons of dry matter per hectare.

#### Establishment of miscanthus in C3 grasslands

The effects of different planting and mowing regimes on miscanthus establishment in grassland and yields in the mixed grassland/miscanthus production systems were assessed in trials established on marginal land near Stuttgart, Germany.

Two field trials were established in May 2012. One on high-productivity (nitrogen-rich) grassland and the other on low-productivity grassland (marginal land, nitrogen-poor soil). A split-split-plot design with four block-replicates was adopted. Each main plot occupied 30.6 m2 and was treated by one of the two establishment regimes (Er1 = cutting the existing grassland vegetation to a height of 5 cm; Er4 = Er1 + spraying herbicide in strips of 20-cm width with a distance of 0.71 m between strips). The secondary treatments consisted of three different cutting frequencies (one, two or three biomass harvests per growing season), which were applied to the 10.2 m^2^ sub-plots within each main plot, starting from the second growing season. In each sub-plot, three different genotypes of *Miscanthus sacchariflorus* (*M. sacchariflorus*) were planted. Additionally, in each treatment, one sub-sub-plot of grassland without miscanthus planted was used as a control for biomass yield comparisons. In addition to these three main genotypes, one standard *Miscanthus sinensis* (*M. sinensis*) clone “Goliath” and one more *M. sacchariflorus* genotype were included in the trials.

The following factors were assessed: Miscanthus plant mortality; miscanthus and grass biomass yield; and a number of phenotypic traits reflecting miscanthus growth and development.

To address biodiversity issues, a vegetation analysis was performed in the two trials established at the university's experimental station: In 2012, before planting the miscanthus, and in 2016, 4 years after planting. In particular, species abundance (according to a multilevel cover-abundance scale; van der Maarel, 2007), species richness (the number of species present) and total canopy cover were recorded for every sub-sub-plot.

### Work package 5: commercial-scale trial and pelleting

The objective of WP5 was to provide data on large-scale miscanthus production for cost assessment and LCA in WP7, to demonstrate large-scale establishment of seed-derived plugs and to identify optimized harvesting and pelleting technologies.

In UK, a large-scale trial was established in 2012 in a marginal field at Blankney, Lincoln. Four replicate plots of 0.25 ha were planted with four seed-based hybrids and the clone *M*. × *giganteus* (resulting in 20 plots, with an area of 5 ha). Over-winter survival rates were 97% for the plug plants, higher than for rhizome-propagated *M*. × *giganteus*. In the second and third years, subplots (sets of rows) were used to investigate herbicide treatments for weed control.

In Germany, a large-scale trial was planted in 2013 at Ihinger Hof field station near Stuttgart with 0.6 ha of the *M. sinensis* population hybrid OPM-111, using the “Checci & Magli” four-row plug planter. Strip plots were used to assess herbicide treatments to improve weed control in miscanthus.

In Ukraine, a large-scale trial was established in 2013 at German Agrarian Center (DAZ) in Potash with three replicates using plugs of OPM-111 and OPM-112 and rhizomes of *M. x giganteus*. In total 23,593 seedlings and rhizomes were planted on a total area of 1.26 ha. Plant losses after transplanting and in first winter were less than 5%.

Yield and quality traits were determined at each site and in the spring following the previous growing season. Additionally, in the UK harvesting techniques (direct chipping and mowing and baling) were compared for speed, yield and quality parameters in the spring following the third growing season. Harvested samples were used for pelleting trials to measure energy requirements at each biomass-formatting step needed to create pellets.

Data from these large-scale trials, combined with commercial knowledge of *M*. × *giganteus* from the company Terravesta, were used to assess the costs and benefits of these methods on the environment and economics of growing the crop (WP7).

### Work package 6: composition and high-value products

The main goal of WP6 was to identify high-yielding miscanthus genotypes with biomass qualities suited to different biobased products. End-use applications assessed included bioethanol, biogas, combustion, and fibreboards.

Data from sequential harvests in the multiple-locations trials in Germany, Russia, and Turkey (WP4) were used to model the quality and yield development of different genotypes at different harvest times.

#### Analysis of combustion quality

The autumn and spring harvests of the third and fourth year stands of the multi-location trials (WP4), as well as the biomass from sequential harvests from the multiple-locations trials in Germany, Russia, and Turkey (WP4) were analyzed for quality parameters relevant for combustion. These include the contents of ash, potassium (K), chloride (Cl), phosphorus (P), and nitrogen (N). Analytical methods are described by Iqbal and Lewandowski (2016).

#### Cell wall analysis and biogas potential

The plant cell wall composition of all genotypes used in OPTIMISC was analyzed in various experiments and correlations with the quality for different biobased applications were evaluated. Three new field trials were established to study the interplay between cell wall composition and saccharification yield as a measure of bioethanol production (van der Weijde et al., 2016a, 2017), combustion quality (van der Weijde et al., 2017), biogas yield (Kiesel and Lewandowski, 2017; van der Weijde et al., 2017), and cutting tolerance of the different miscanthus genotypes (Kiesel and Lewandowski, 2017). Additionally the effects of abiotic stresses and geographic location on biomass quality were studied using the material harvested in the multi-location trials (WP4) and the abiotic stress tests (WP3) (van der Weijde et al., 2016b).

#### Chlorophyll and protein extraction

The chlorophyll and protein production potential of the stay-green OPM-111 *M. sinensis* hybrid planted at all three large-scale trial locations (WP5) was quantified for in-season and end-season harvests. Chlorophyll was extracted using the Soxhlet method. Total protein analysis was performed according to the Kjeldahl protocol.

### Work package 7: modelling (yield, LCA, costs)

In this work package, the MiscanFor model was extended to create a European miscanthus yield and biomass potential model for diverse genotypes. The MiscanFor model was originally developed using experimental data available in 2008 from multi-location trials with the only commercially planted miscanthus clone *M*. × *giganteus*. In the third and fourth growing seasons after planting the WP4 multi-location trials, regular measurements were taken to quantify “in season” growth curves for green leaf area index, radiation intercepted by the canopy and standing biomass. New parameters for process descriptions between thermal time (degree days) and green leaf area index; and accumulated radiation intercepted and yield were derived from ten genotypes from 4 out of the 6 locations. These new growth and climate data sets expand those currently available for *M. x giganteus*. These data improve the model parametrization for *M*. × *giganteus* over a wider climatic range and extend the model to include a range of germplasm types and novel hybrids with commercially relevant traits.

A cost and life cycle assessment (LCA) was performed for seven selected miscanthus-based product chains. For this purpose, data from field trials in WP4 and 5 were used. The LCA was performed using the Gabi 5 Software. The overall biomass transport distance was assumed to be 400 km when bales were transported to the bioethanol plant or to the plant producing insulation material as well as in the value chain “Combined heat and power (CHP) bales.” For the value chains “CHP pellets” and “Heat pellets” the bales were transported 100 km to a pelleting plant and from there the pellets were transported 400 km to the power plants. The average farm-to-field distance was assumed to be 2 km. This transport distance is also assumed for the value chain “heat chips” in which a utilization of the chips as a biomass fuel on the producing farm was assumed. Because of the higher biomass requirements of the biogas plant an average transport distance of 15 km from field to plant was assumed.

## Results and discussion

The results of the various research activities are summarized in Table 5. These are then discussed, focusing on their relevance for the advancement of miscanthus and implementation of miscanthus-based value chains.

**Table 5:** **Overview of development needs for miscanthus, how these were approached and relevant results**.

	**Development need**	**Development approaches**	**Main results**
Growing the crop	Adaptation to different climatic conditions and to adverse and marginal site conditions	Provision (WP2) and evaluation of new breeding material (WP3, 4, 5, 6)	More than 160 miscanthus genotypes were provided for screening under field and controlled conditions. *M. sinensis* is more difficult to *in vitro* culture than *M. sacchariflorus* and their hybrids. Improvements in *in vitro* tillering methods included new surface sterilization approaches for a rhizome, node and flower meristems. Protocol adaptation and persistence achieved > 70% success rate for transfer of germplasm to *in vitro*.
		Better understanding of genotype x environment interactions (WP4, 5)	Recommendations for optimal choice of genotypes for all European regions.
			Northern Europe: OPM-08, -06, -10, -09
			Central Europe: OPM-09, -10, -06, -03
			Southern Europe: OPM-11, -14, -02, -03
		Develop chilling and frost tolerant genotypes (WP3) to: a) Extend productive range of miscanthus to the north and east b) Improve establishment and overwintering success c) Breed genotypes with a longer growing season	Genotypes identified with relative tolerance to chilling and frost and with high early vigor, which have potential for cultivation in regions further north and east and as starting material for breeding.
			*M. sinensis* and *M. sinensis x sacchariflorus* hybrid genotypes were more frost tolerant than *M. sacchariflorus genotypes and M. x giganteus*.
		Develop water-use efficient and water-stress tolerant genotypes (WP3) to: a) Extend the productive range for miscanthus further south b) Provide genotypes for marginal land	*M. × giganteus* has medium tolerance in terms of maintaining biomass production under drought, but recovers well when water is re-applied.
			Several genotypes were identified with improved yield compared to *M. × giganteus* under water-limiting conditions and with improved recovery potential after drought.
			A few genotypes are very high yielding under drought conditions despite only having medium drought tolerance. These genotypes may not perform so well under continuous drought. Of 7 genotypes with drought yields significantly higher than *M. x giganteus*, only 3 are in the top 10 in terms of drought tolerance. These may be suited to more southerly locations.
			Drought tolerance mechanisms include reduced water loss, such as leaf rolling, and water seeking strategies such as increased root to shoot ratio.
		Develop salinity-tolerant genotypes (WP3) for marginal land	Genotypes identified with high yields under both optimal and saline conditions.
			Starting material for breeding for salt tolerance through improved ion-exclusion activity.
			*M. sacchariflorus* and *M. sinensis* genotypes show salinity tolerance through mechanism of salt exclusion.
			Land areas with soil electric conductivity (EC) up 2.5 S/m suitable for miscanthus production.
		Develop establishment methods for marginal land and grasslands	In Germany, 80% establishment success rate for miscanthus into C3 grassland was achieved with both a no-till method and conventional pre-planting disturbance (i.e. mowing or herbicide spraying applied before planting miscanthus).
			Competitive miscanthus genotypes with tall, thick shoots to be chosen for establishment in grassland.
	Reduction of biomass production costs	Target the development of genotypes that can be established via seeds (WP2, WP5)	Commercially scalable protocols for plug planting seed-based hybrids were developed. (The project produced 100,000 plants needed for large-scale trials in three locations: UK, Germany and Ukraine).
		Identify more winter-hardy genotypes to reduce or avoid over-winter losses (WP3)	See above
		Reduce the input demands, e.g. nitrogen fertilization, of biomass production	As expected, significantly lower nutrient offtake in early senescing genotypes. This reduces the fertilizer offtake and increases biomass quality when used for heat production. Unexpectedly, leaf share not always linked to offtakes at harvest.
	Improvement of yield and biomass supply stability	Identify high-yielding genotypes adapted to different climatic conditions (WP4)	Several genotypes were identified with high yields (exceeding that of *M. × giganteus*) under different climatic conditions. In particular, OPTIMISC has helped identify genotypes suitable for cultivation in climatic extremes: in colder climates (Moscow), in hot climates with low water availability (Adana) and on marginal land (Aberystwyth).
		Increase yields of valuable biomass co-products (WP5, 6)	Chlorophyll and protein can be extracted before biomass goes to biogas production.
Harvesting	Reduction of harvest and logistic costs	Reduce harvest, logistic and drying costs by selection of genotypes with dry biomass at harvest (WP4, 5). Reduce pre- and post-harvest losses (WP 5)	Direct chipping with a 7.5-m cutter on a self-propelled forage harvester was the most time-efficient cutting method. However, in climates with mild winters and inadequate senescence, the indirect mowing and baling methods are more scalable due to more efficient transport and storage.
	Optimization of harvest time in terms of quality and reduction of harvest losses	Select genotypes with improved senescence patterns for dry harvestable biomass (WP4, 6)	Significant GxE (Genetic x Environment) interaction for senescence was observed. The interspecies hybrids tested senesced earlier than wild types.
Connecting to market	Biomass quality suitable for purpose of user	Understand genetic variation and effect of drought on biomass quality performance (WP 3, 6)	GxE interaction for biomass quality relevant for combustion and production of ethanol and biogas.
			Drought has a negative effect on yield but a positive effect on biomass quality. Developing drought resistant genotypes would create opportunities for growing high-quality miscanthus biomass on marginal soils (in particular dry areas).
		Diversity in biomass quality of miscanthus genotypes	There are large differences in biomass quality, and consequently performance in different chains, e.g. bioethanol and biogas, among miscanthus genotypes.
			Many genotypes have been identified with better biomass quality than *M. × giganteus*.
	Development of novel value chains	Biogas production was identified as a promising value chain for miscanthus biomass (WP6). *Miscanthus × giganteus* and novel genotypes showed high and promising potential.	October was identified as optimum biomass harvest date for Central Europe due to a very high biogas potential and sufficient cutting tolerance.
			Novel genotypes showed significantly higher specific biogas/methane yield (up to 520 ml/g DM) than *M. × giganteus*.
	Optimization of biomass supply chain	Develop logistics for the supply of transportable, storable and tradeable biomass (WP5)	Shorter hybrids with thinner stems had the benefits of lower moisture content (13%), higher bale weights (500 kg for *M. × giganteus*, vs. 650 kg) with less string breakages and ca. 20% power to pellet. However, compared to *M. × giganteus*, lower yielding and the pellets are 5% less dense.
			Pellets: highest bulk density for *M. × giganteus* biomass (OPM-09) at 810 g/l and the lowest for OPM-12 at 664 g/l.
			All miscanthus genotypes can be pelleted. *M. × giganteus* most difficult to pellet due to hard, stiff stems. *M. sinensis* OPM-12 best to pellet genotype.
			Pelleting costs 40–80 Euro/ton pellets.
	Optimization of miscanthus-based product chains	Identify cost-optimized and environmentally benign miscanthus-based product chains (WP7).	Up to 25 t (small-scale combustion, chips) and 31 t (insulation material) CO_2eq_./ha^*^a savings.
			In Central Europe cost of fuel for domestic small-scale combustion (≤ 2 ct/kWth) compete well with other fuels.
			Lowest carbon mitigation costs of -78 Euro/t CO_2eq_. avoided for local small-scale combustion of chips.

*These are listed from top to bottom along the production to utilization chain*.

### Options for producing miscanthus in different climates and on marginal land

This section presents the results of testing novel miscanthus germplasm in comparison to *M*. × *giganteus* over a wide range of European climates and also recommendations for miscanthus production on marginal land.

#### Yield performance of novel miscanthus genotypes over a wide range of climatic conditions in Europe, Ukraine, Russia, and Turkey

Some of the trial locations represented marginal (limiting) growth conditions for miscanthus. In particular, the field trial in Adana (Turkey) exhibited the highest air temperature and driest soil conditions among the OPTIMISC experiments, and the trial in Moscow (Russia) represented the coldest location with cold winter temperatures, late spring frosts and longer summer photoperiod than at the other sites. The Moscow site also suffered a summer drought in 2014.

As part of the OPTIMISC multi-location trial (WP4), 15 genotypes were planted on a marginal land site at Aberystwyth (UK). This field site was formally grassland, with low nutrient levels and shallow soils. Additionally, the growing season temperatures and radiation levels in cool wet summers here delay establishment rates. The shallow soils lead to rapid changes in soil moisture levels, with flooding conditions after rainfall and drought stress in summer. The high stone content of the soil made miscanthus establishment difficult.

The more challenging growth conditions at these sites resulted in lower miscanthus yields at Aberystwyth and Moscow than at the other four locations. The drought at the Mediterranean site Adana caused the miscanthus to start senescing in July and therefore only dry matter (DM) yields of up to 15 t/ha^*^a could be harvested here. The highest DM yields (up to 20 t/ha^*^a harvestable biomass in early spring) were achieved at Potash/Ukraine, where good clay-rich soils and good water supply prevailed. The south German site Stuttgart is characterized by low soil depth (on average 60 cm soil horizon) and encountered a drought in summer 2015. It was only possible to harvest up to 18 t DM/ha^*^a here.

Apart from the Ukrainian site Potash, miscanthus genotypes with yields exceeding that of *M*. × *giganteus* (OPM-09) were identified at all sites. These were either other *M. sinensis* × *M. sacchariflorus* hybrids (Moscow, Aberythwyth, Wageningen, Stuttgart), *M. sacchariflorus* (OPM-02, Stuttgart) or *M. sinensis* (Adana).

We therefore conclude that new genotypes are available that can out-perform *M*. × *giganteus*, especially on marginal lands.

Table 6 gives a ranking of genotypes according to yield and yield stability. Both absolute yield (top panel) and yield stability (lower panel) are important factors in selection.

**Table 6:** **Yield ranking across the six sites, in the first 3 years (spring harvest years) after planting miscanthus**.

**Largest biomass yield (mean yield across three plots)**	**2013**	**2014**	**2015**
Best Yield	OPM-09	OMP-06	OPM-06
Second Best Yield	OMP-06	OPM-09	OPM-09
Best Yield Adana	OPM-09	OPM-09	OPM-09
Best Yield Stuttgart	OPM-01	OPM-03	OPM-06
Best Yield Potash	OPM-06	OPM-06	OPM-02
Best Yield Wageningen	OPM-06	OPM-09	OPM-08
Best Yield Aberystwyth	OPM-08	OPM-08	OPM-08
Best Yield Moscow	OPM-06	OPM-06	OPM-06
**Least yield variability (Coefficient of Variablility)**	**2013**	**2014**	**2015**
Best CoV	OPM-11	OPM-06	OPM-10
Second Best CoV	OPM-06	OPM-10	OPM-06
Best CoV Adana	OPM-09	OPM-09	OPM-10
Best CoV Stuttgart	OPM-02	OPM-07	OPM-05
Best CoV Potash	OPM-12	OPM-01	OPM-04
Best CoV Wageningen	OPM-04	OPM-15	OPM-13
Best CoV Aberystwyth	OPM-15	OPM-11	OPM-08
Best CoV Moscow	OPM-12	OPM-13	OPM-02

Table 7 gives recommendations for the use of different genotypes, with reasons, based on field observation. *M. sacchariflorus* types are characterized by spreading rhizomes, which can lead to escape of the crop. Overall, *M. sacchariflorus* types tested here are only recommended for southern European sites with irrigation or no susceptibility to drought. *M. sinensis* × *M. sacchariflorus* hybrids, including *M*. × *giganteus*, are recommended for most areas of Europe (OPM-09, OPM-10) or northern Europe (OPM-08), mainly on account of their high yields.

**Table 7:** **Recommendations for the choice of miscanthus genotypes for different European regions**.

**Genotype ID**	**Recommended**	**Reason**
OPM-01 (*M. sac*)	No	Poor yields, spreading (creeping) rhizome.
OPM-02 (*M. sac*)	Sometimes	Only in southern Europe with irrigation where drought possible. Excellent yield but requires high temperatures and susceptible to drought. Has spreading rhizome but can be managed by mowing field plot borders once or twice mid-season.
OPM-03 (*M. sac*)	Sometimes	Mainly in southern Europe with irrigation where drought is possible; also possible for Central Europe. High yielding in some locations. It has a spreading rhizome but can be managed by mowing field plot borders once or twice mid-season.
OPM-4 (*M. sac*)	No	Poor yields and a spreading rhizome.
OPM-05 (*M. sac × M. sin*)	No	Acceptable yield but out-performed by similar hybrids.
OPM-06 (*M. sac × M. sin*)	Yes	Central and eastern parts of northern Europe. Excellent yields but lodging crop not acceptable to farmers.
OPM-07 (*M. sac × M. sin*)	No	Poor yields.
OPM-08 (*M. sac × M. sin*)	Yes	Northern Europe. Excellent yields at the cooler sites.
OPM-09 (*M. x gig*)	Yes	Most of Europe. Excellent yields generally sufficient for large areas of Europe, especially with the projected climate changes of warmer wetter winters, which is consistent with the years these trials were conducted. Limited by clonal propagation.
OPM-10 (*M. sac × M. sin*)	Yes	Most of Europe. Excellent yields and low moisture content at harvest on account of early senescence.
OPM-11 (*M. sin*)	Yes	Southern Europe. Good yields at the locations with warm summers and frequent droughts (this clone did not perform well in Aberystwyth or Moscow).
OPM-12 (*M. sin*)	No	This seeded germplasm entry is heterogeneous. It flowers too early to attain high yields. It produces viable seeds. (In the WP4 trials, establishment problems were largely linked to logistical issues around planting, rather than being a reflection of germplasm establishment ability.)
OPM-13 (*M. sin*)	Yes	Potential in areas with warm summers and drought. Advantages: seed-based and non-creeping. More homogeneous than OPM-12 and OPM-15. Generally lower yielding than interspecies hybrids. It was less susceptible to drought conditions in Turkey.
OPM-14 (*M. sin*)	Yes	Southern Europe. Similar to OPM-13, but on average slightly lower yielding.
OPM-15 (*M. sin*)	No	As for OPM-12. This seeded germplasm entry is heterogeneous. It flowers too early to attain high yields. It produces viable seeds.

The *M. sinensis* type OPM-11 is recommended here for Mediterranean areas, where it can make best use of the spring period before the onset of summer droughts (Table 7).

**Figure 2:**
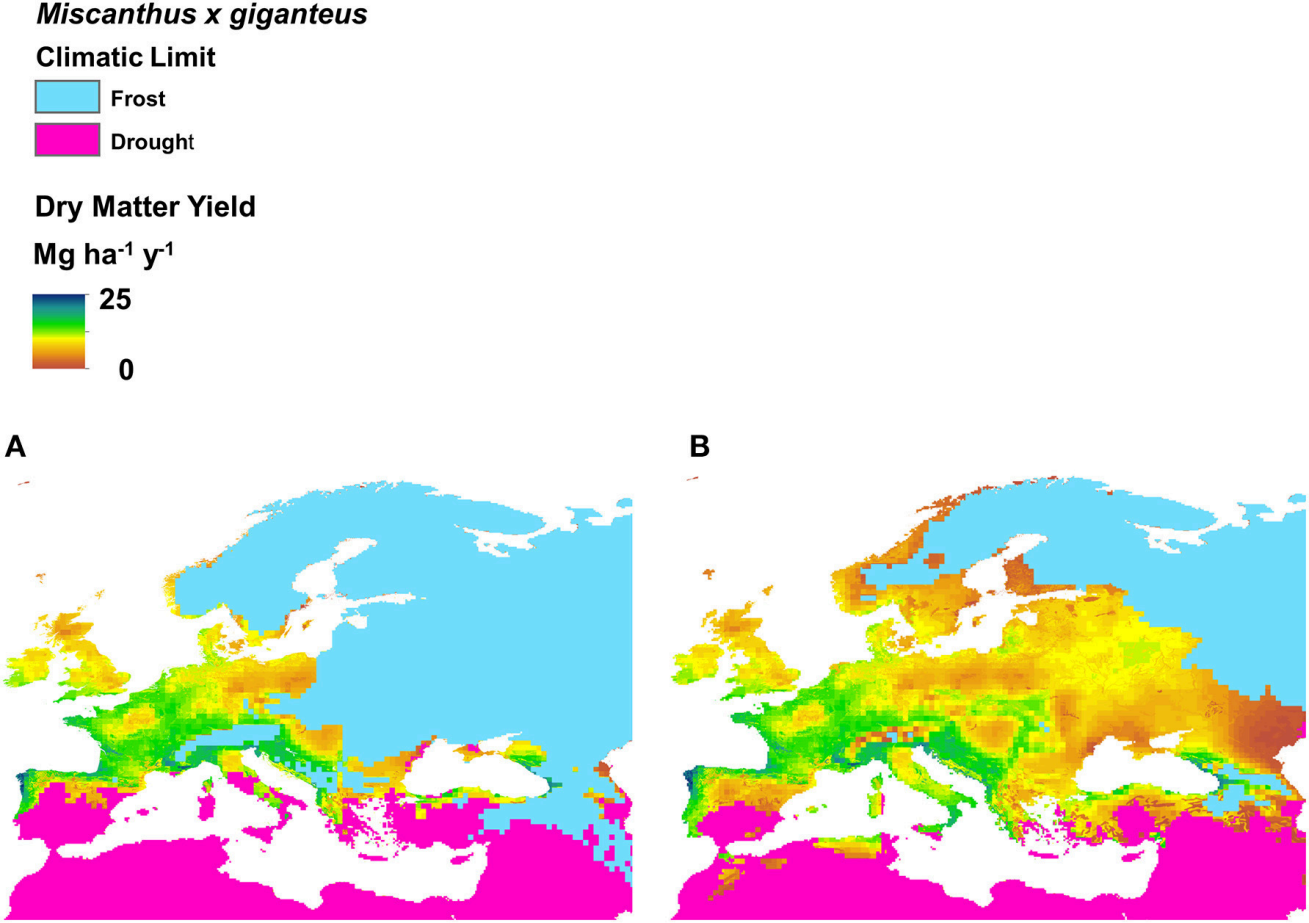
**Bioclimatic envelope of *Miscanthus* × *giganteus* showing limit of frost and drought tolerance**. Excluded area is shown in light gray. Left **(A)** shows the original Hastings et al. (2009) bioclimatic envelope and right **(B)** shows the revised estimation of the bioclimatic envelope for *M*. × *giganteus* and the new trialed hybrids resulting from the research in this project. The crop yield prediction for *M*. × *giganteus* is displayed on a scale from 41 Mg ha^−1^ (black) to 0 Mg ha^−1^ (gray). Both bioclimatic envelopes are based on recent climate data (2000 to 2009) and FAO/IGBP plant-available water estimates on a 5-min grid. The new cold limit considers the data from in-field soil temperature measurements and the overwinter survival success. The new drought limit is based on observed in-field drought responses and water balances with estimates of plant-available water derived from depth and soil textures measurements. This high-level analysis does not identify the marginal lands within the grids where the yields may be lower than those indicated.

The potential miscanthus growing area for Europe was modeled based on measurements of field performance of the genotypes OPM-01 to OPM-15. Compared to a scenario where only the genotype *M*. × *giganteus* is available (Figure 2A), a large potential expansion of the miscanthus growing area to the east, south and north of Europe is predicted for a scenario where the genotypes screened in OPTIMISC can be grown commercially (Figure 2B).

#### Identification of stress-tolerant miscanthus genotypes

One important result from the OPTIMISC project is the expansion of the potential miscanthus production area in Europe (as shown in Figure 2). This is achieved mainly by the successful identification of stress-tolerant genotypes for biophysically marginal cultivation conditions in WP3. Biophysical marginality is often caused by the abiotic stresses of water shortage, unfavorable temperature or poor soil conditions, including salinity.

The evaluation of stress tolerance in plants is not straightforward, as it is strongly affected by environmental conditions. Therefore, we focused on finding relevant traits and mechanisms for four abiotic stressors that are relevant to miscanthus cultivation (drought, salinity, chilling, and frost), assessing genetic diversity in a range of cultivars and breeding material, and identifying traits that can be used for selection and improvement of miscanthus cultivation on marginal lands. At the same time, genotypes were selected that are expected to have a relatively high production under marginal conditions where they experience drought, salt, chilling, and/or frost stress.

#### Drought

The response to drought and recovery after drought differs between and within species. Recovery potential is likely to be of critical importance for yield under conditions with regular drought spells. As this is a likely climate-change scenario, recovery should be part of any drought tolerance evaluation for miscanthus.

Among the genotypes tested, some produced high biomass yield under both well-watered and drought conditions. Other genotypes were not high yielding under well-watered conditions, but showed only a small reduction in yield under limited water availability. While it is tempting to speculate that these would be potential sources of drought-tolerance traits to be utilized in breeding programs, it is important to exclude genotypes that require less water simply due to their small size and slow growth.

Several of the genotypes screened demonstrated a harvestable biomass yield greater than that of the standard *M*. × *giganteus* (OPM-09). Four of the genotypes that produced more biomass than *M*. × *giganteus* under control conditions were also among the most drought-tolerant genotypes (maintaining a high percentage biomass under drought): OPM-06 (hybrid), OPM-25 (*M. sacchariflorus*), OPM-77 (*M. sinensis*), and OPM-27 (*M. sacchariflorus*). A further 10 genotypes showed medium tolerance (OPM-05 (hybrid), OPM-86 (*M. sinensis*), OPM-38 (*M. sinensis*), OPM-69 (*M. sinensis*), OPM-02 (*M. sacchariflorus*), OPM-19 (*M. sacchariflorus*), OPM-20 (hybrid), OPM-23 (*M. sacchariflorus*), OPM-07 (hybrid), and OPM-39 (*M. sinensis*) and also exceeded *M*. × *giganteus* yield under control conditions. These 14 genotypes had higher yields than *M*. × *giganteus* in both drought and well-watered conditions. A single drought-susceptible genotype [OPM-50 (*M. sinensis*)] yielded more than *M*. × *giganteus* when watered but not under drought, emphasizing the importance of biomass yield *per se* as opposed to maintaining biomass yield in a smaller plant. Of the 10 highly tolerant genotypes (in terms of maintained biomass yield), 5 also demonstrated relatively high maintained soil moisture. This indicates that these plants are water-use efficient and are able to maintain biomass production without depleting soil moisture. Of the 14 genotypes that outperformed *M*. × *giganteus* under control and drought conditions, five were *M. sacchariflorus*, five were *M. sinensis* and four were hybrids (van der Weijde et al., 2016b).

It should be noted that different traits may be of more or less importance depending on the timing and severity of the drought stress, and that it is a combination of traits that provided tolerance under the conditions applied in this experiment. While growth cessation and damage protection may be good strategies to withstand the adverse effects of a relatively short but severe drought, long-term mild droughts were not tested in this study. It remains to be seen whether the same genotypes are productive under such conditions, or whether traits enabling the maintenance of growth may be more favorable.

#### Salinity

Saline soils affect crops in two ways: It induces water shortage due to osmotic stress and accumulation of salt in the plant can have toxic effects.

In our screen, we found indications that miscanthus uses two mechanisms to mitigate the effects of salinity. The best performing genotype (OPM-56, *M. sinensis*) utilizes a mechanism that actively keeps the ions from accumulating in the leaves, thus minimizing damage to essential physiological processes like photosynthesis. This mechanism is known as salt exclusion, and is known to be able to confer salt tolerance to rice and wheat (Munns et al., 2012). The causal gene in these two cereal crops is HKT1; 5, an ion transporter that takes Na^+^ out of the xylem and into the parenchyma cells in the roots, avoiding Na^+^ accumulation in the leaves. This is a strategy that can be effectively selected for by measuring ion contents in the leaves of plants. In addition, it would be interesting to target the HKT1; 5 gene in miscanthus as the causal gene for this mechanism. Further exploration in miscanthus germplasm to identify the most effective alleles of this gene and for the Na^+^ exclusion mechanism is therefore recommended. In view of the quality of harvestable yield, the salt exclusion mechanism may also be preferred. High concentrations of ions are known to interfere with combustion quality, and may be a problem for saline cultivation of miscanthus. Improving salinity tolerance by improving salt exclusion properties enhances yield under saline conditions, and at the same time improves product quality.

Field trials were performed with the genotypes *M*. × *giganteus* (OPM-09), OPM-01, -03, -06, -08, and several *M. sacchariflorus* genotypes, selected from marginal and saline land in North-East China. The trials revealed that *M*. × *giganteus* is not suitable for saline land. Different *M. sacchariflorus* genotypes proved salinity-tolerant. The yield declined with increasing soil salt electrical conductivity (EC) values. A soil EC value under 2.5 had little effect on yield, but at a soil EC above 3 yields decline dramatically. Compared to slightly saline land (average EC of 1.10) the yields of the most salinity- tolerant genotypes on the heavily saline site (average EC of 3.85) declined by 30–55% in the second stand year.

In conclusion, the highest-yielding genotypes under controlled conditions (especially *M. sinensis* OPM-56) have potential to grow in saline soils, and should be tested under field conditions. In addition, several of the *M. sacchariflorus* genotypes tested in the field can be recommended for growth under saline soil. Land areas with a soil EC value up to 2.5 are suitable for miscanthus production.

#### Low temperature

A small number of genotypes were analyzed for photosynthetic and biochemical traits, which are likely to be linked to chilling tolerance. These revealed large variations for both trait types (Mortaignie, 2014; Fonteyne et al., 2016a). This indicates that a combination of these traits may in fact enhance chilling tolerance and can be targeted for combined selection (Fonteyne et al., 2016a). Outdoor evaluation of chilling tolerance indicated a wide variation in the germplasm and that emergence of first shoots, time to reach 50 cm shoot length and early growth rate are good parameters for large-scale chilling tolerance evaluation.

Frost tolerance evaluation of a set of miscanthus genotypes was performed using potential marker traits such as moisture content, ion leakage and phenological characteristics. None of these markers was strongly correlated to frost tolerance. The best marker trait to determine frost tolerance turned out to be the LT_50_ in artificial rhizome freezing tests. The LT_50_ can be directly related to winter survival (Clifton-Brown and Lewandowski, 2000). Mechanisms underlying freezing tolerance in miscanthus are still elusive, but may be linked to production of specific metabolites and molecules that stabilize cell structures, most notably membranes, under freezing conditions (Thomashow, 1999).

In general, the hybrid genotypes were more frost-tolerant and the *M. sacchariflorus* and *M*. × *giganteus* genotypes were less frost-tolerant. On average, the *M. sacchariflorus* genotypes had a significantly higher LT_50_ than the hybrids, while the *M. sinensis* genotypes were not different from either group, but genotypes with higher frost tolerance than *M*. × *giganteus* were found in all species groups.

#### Stress-tolerant genotypes in the wider context

Based on our observations, the miscanthus genotypes tested under various conditions display a wide range of variation in response to abiotic stresses, but this may not be the full range of tolerance to stresses that can be exploited in miscanthus germplasm. For instance, the salinity field trial in Dongying showed that several of the newly collected Chinese *M. sacchariflorus* genotypes were relatively tolerant to saline conditions (with CN32 being most tolerant), although its tolerance was not much higher than some of the genotypes tested under controlled conditions. Further collection of miscanthus material growing on marginal soils is required, and this should be tested using the screening procedures developed within this project as well as in the field, alongside the best performers selected in this project.

Predicting how tolerant to stresses the selected genotypes will be in terms of water requirements and temperature is not straightforward. The field trials indicate that some genotypes perform better in relatively hot climates, while others thrive even after cold winters. However, the set of genotypes tested in the multi-location trials was too small and not enough of the best-performing genotypes were tested under field conditions. Thus, the logical next step would be to test the top performers from the controlled condition evaluations in different climatic regions to establish whether these selections are also relatively tolerant under varying field conditions.

For salinity at least, it can be deduced that the best-performing miscanthus genotypes' tolerance of saline soils is higher than in cereals, even barley (considered to be a salt-tolerant cereal). This offers opportunities for miscanthus cultivation in marginal, saline areas.

We would recommend that genotypes with extreme traits are crossed into highly productive parental lines, and the progeny are evaluated for resilience in further laboratory screens and field trials. Identification of the trait variation is an important step, but only one of many steps necessary for genetic improvement. This is part of a longer-term program of breeding and evaluation, which needs ongoing public support to deliver the resilient hybrids required to drive the feedstock supply for the bioeconomy.

#### Methods for the establishment of miscanthus on marginal land

Challenging establishment conditions, including drought, stoniness, and low temperature, present a major barrier to miscanthus production on marginal land (Xue et al., 2016). The OPTIMISC project developed technical approaches for the establishment of miscanthus under marginal soil conditions (WP5) and on grassland (WP4).

The planting of seed-derived plugs proved to be most successful method for miscanthus establishment on marginal soils. Covering the plants with a plastic film accelerates their growth. The film keeps the humidity in the topsoil and increases the temperature. This is beneficial for the plants, especially on light soils with a higher risk for drought stress and in cool temperatures.

In Europe, there are large areas of marginal land covered by grassland. The OPTIMISC project performed field trials for the establishment of miscanthus into grassland (WP4). The hypothesis was that the inclusion of miscanthus (high-yielding C4 grass species) into C3 grasslands could be beneficial for biomass yield, given that suitable miscanthus genotypes are to be carefully selected for this purpose. Examples of yield increase in C3/C4 mixed grasslands compared to pure C3 grasslands can be found in the scientific literature (Adler and Sanderson, 2009). Growth patterns of C3 and C4 grasses are often complementary and lead to higher total annual harvestable yield (Thumm et al., 2012). Addition of miscanthus into C3 grasslands in temperate climates could also improve biomass quality for certain purposes, such as combustion.

The establishment of miscanthus on grassland proved successful with two propagation techniques: (1) direct planting of rhizomes in the soil and (2) transplanting of pre-grown, rhizome-derived plantlets. The second technique appeared to lead to better establishment success, although this depended on the genotype.

Pre-treatment of the existing vegetation is important to ensure good establishment of the introduced miscanthus plants. Cutting the existing vegetation and spraying herbicide in narrow strips (defined as intermediate in severity) appears to be the most advantageous pre-treatment of the grassland. This improves miscanthus establishment without negatively impacting on the productivity and existing vegetation of the C3 grassland itself.

Strong, competitive miscanthus genotypes with tall, thick shoots seem to be a better choice for establishment on grassland than genotypes with short, thin shoots, regardless of the species.

The C3/C4 grasslands can and should be managed by multiple in-season mowing of green biomass, as is usually performed on European grasslands. Our results demonstrated that a mowing regime with two harvests per year (spring and autumn) is most suitable to achieve good biomass yields from these mixed grasslands. Harvesting once per season in autumn leads to a higher proportion of miscanthus biomass but to a lower biomass gain from the C3 grassland due to its natural senescence early in summer.

#### Meeting biodiversity concerns

Biodiversity issues need to be considered when planting miscanthus into C3 grasslands. In our trials, vegetation analyses performed before and 3 years after the establishment of miscanthus revealed that the species richness and abundance did not change significantly with this addition. However, the miscanthus was planted at a relatively low density and remained only a small contributor to the plant canopy and biomass (3–6%) due to high competition. Planting at higher densities or development of the miscanthus over time could potentially bring about changes in the existing plant communities.

As miscanthus is a not native to Europe, there are also concerns about uncontrolled spreading of this crop. There are two potentially relevant pathways for such spreading: (1) via creeping rhizomes and (2) via seed.

Creeping rhizomes were observed in several *M. sacchariflorus* genotypes, one of which was strongly creeping. We therefore recommend excluding genotypes with this feature from commercialization (see Table 7).

Germination tests carried out under controlled conditions showed that 10 of the 15 miscanthus genotypes tested in the OPTIMISC multi-location trials produced viable seeds. All these genotypes belonged either to *M. sinensis* species or *M. sinensis x M. sacchariflorus* hybrids. The highest seed germination rates were observed in Germany and the Netherlands and the lowest in the most southerly trial location of Turkey and two more northerly (colder) sites in Russia and Ukraine. The germination rate was especially low (on average 0.2 ± 0.13 seeds per panicle in 2014) in Russia (Moscow area), where long-day conditions retarded the transition to flowering and the vegetation period is short, preventing complete seed ripening (plant senescence occurs earlier). Strong genotypic differences were observed for seed germination. Two *M. sinensis* genotypes/accessions (OPM-12 and OPM-13) showed particularly high numbers of viable seeds per panicle (on average 150 ± 38 and 123 ± 34 seeds per panicle, respectively, in 2014). The *M. sacchariflorus* genotypes produced no viable seeds at all six trial locations. The *M. sinensis x sacchariflorus* hybrids (OPM-05–OPM-10) showed an average (six locations pooled, 2014) of 38% lower seed germination per panicle than the *M. sinensis* accessions. This ratio varied however between locations and genotypes. In the UK for example, the number of germinating seeds per panicle was approximately 50% higher in the hybrids than in the *M. sinensis* accessions. By contrast, in Germany, Ukraine and Turkey, this number was much lower in the hybrids than in *M. sinensis*. The highest number of geminating seeds per panicle was observed for the genotypes OPM-05 and OPM-10 (all locations pooled, in 2014).

Spreading via seeds was carefully monitored in the OPTIMISC trials. Volunteer miscanthus seedlings were found at two of the six locations of multi-location trials (WP4), the Netherlands and Germany. These seedlings were found outside the planted plots but within the plantation borders. No accidental spreading via seeds was observed at any of the more southerly or more northerly locations. In the south, seed germination in the field was possibly prevented by drought conditions, in the north by low temperatures and a shorter vegetation period. No volunteer miscanthus seedlings were found outside the plantation borders.

From these observations, we conclude that spreading via seeds in miscanthus—relevant for *M. sinensis* and *M. sinensis* × *M. sacchariflorus* hybrids—can be prevented by careful choice of genotype. Therefore, genotypes should be recommended that either do not form fertile seeds or that are unable to establish via seed due to the climatic conditions of a specific site.

Another biodiversity concern is that miscanthus, as a perennial crop with tall and dense stands, may give rise to a monoculture, which supports only low species diversity.

Our results show that young miscanthus stands sustain high plant species diversity before the canopy closure. Species richness was found to correlate negatively with the density of the stands and to be lower in mature plantations. However, even the 16-year-old, dense miscanthus plantations supported up to 16 different weed species per 25-m^2^ plot, accounting for up to 12% of the plantation. The literature data support this finding: Miscanthus stands are usually reported to support farm biodiversity, providing habitat for birds, insects, and small mammals (Semere and Slater, 2007a; Bellamy et al., 2009). Studies by Semere and Slater (2007b) have shown biodiversity in miscanthus to be higher than in other crop stands, but still lower than in open field margins.

### Scaling up miscanthus production and connecting to markets

The results of the OPTMISC project can contribute to the fulfillment of requirements for scaling up miscanthus production by:

– Providing seed-based, low-cost, and safe establishment methods;
– Providing germplasm for the development of stress-tolerant miscanthus varieties, adapted to a wide range of climatic conditions in Europe;
– Providing higher and more stable-yielding miscanthus genotypes that can also be produced economically on marginal lands;
– Developing genotypes that are optimally suited to harvesting, processing and biomass user requirements;
– Developing harvesting and densification technologies;
– Improving the marketability of miscanthus biomass by assessing new miscanthus-based value chains and demonstrating how the biomass can be suited to user requirements.

#### Seed-based establishment methods

**Figure 3:**
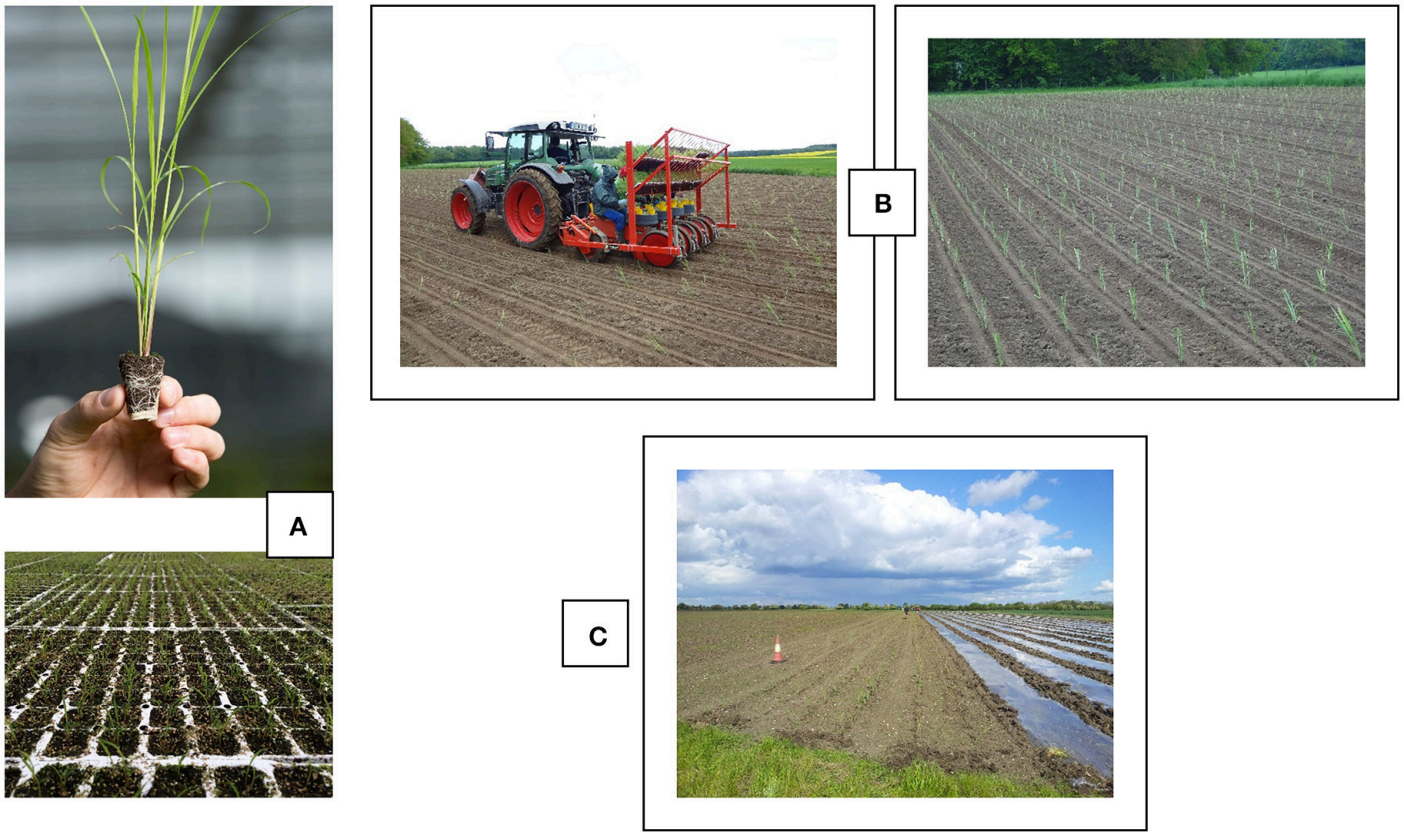
**(A)** Miscanthus establishment starting with seeds sown in modules (plugs) and grown in the greenhouse. **(B)** A Checci & Magli planter in action and planted field. **(C)** Film technology protects modules from drying out and provides them with extra heat units.

Cloning is expensive and the process of upscaling to the large areas necessary to deliver sufficient biomass for a future European bioeconomy would be too slow. For this reason, four seeded hybrids were included in the WP4 and WP5 trials. Although these were not as productive as the interspecific hybrids [including *M*. × *giganteus* (OPM-9)] and therefore not commercially “recommended,” we have pioneered the upscaling of the planting of seed-based hybrids using plugs. These plugs are also called “modules” and were originally developed for the vegetable industry. Seeds are sown by machine and raised in the greenhouse (Figure 3A) before being planted out in the field (Figure 3B). It is anticipated that seed-based establishment methods will prove most effective for the scaling up of miscanthus production because they have the following advantages:

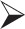
 With increasing market demand, large quantities can easily be provided, once seed production has been well developed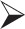 Short growing period for plantlets: Only 8–10 weeks from seed to final product (plugs)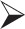 Plug production is energy efficient (no need for refrigerators)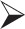 Low establishment costs

When establishing miscanthus via seed in temperate climates, it is recommended that newly planted stands are protected with plastic film (Figure 3C) as this increases establishment success and it is anticipated that it can reduce the length of the establishment period so that an economic biomass yield is produced earlier.

However, if seeds cross out or are not genetically uniform, inhomogeneous field stands are possible.

During the term of the OPTIMISC project, major advances in breeding interspecific hybrids have been made in the UK (Clifton-Brown et al., 2017). The next steps in this development include determining how to: (1) increase the seed production potential of elite interspecific crosses; 2) optimize planting density; (3) maintain effective weed control during establishment—especially where the crop is to be established on marginal land.

If investment in breeding and trialing is sustained, we expect to be able to apply the knowledge gained from these parallel roads of development to achieve commercial upscaling by about 2020.

#### Genotypes suitable for processing and use—biomass quality

The properties of miscanthus biomass determine its harvestability, transportability, marketability, and usability. Moisture content must be appropriate for harvest technology and storage. If the moisture content exceeds 20%, there is a danger of self-ignition of the biomass during storage. For ensiling, the water content should ideally be in the range of 65–72%. The combustion quality of biomass is determined by both water content and the concentration of elements that cause corrosion and reduce the ash melting point, mainly chloride (Cl), potassium (K), and nitrogen (N) (Lewandowski and Kauter, 2003). Densification of biomass, for example in the form of bales or pellets, is often necessary for storage or long-distance transport. (The economic relevance of this is discussed in Section Miscanthus value chains—options and implementation). The organic composition of the cell walls affects the digestibility of the biomass and therefore determines its usability in ethanol or methane (biogas) production.

The OPTIMISC project found in WP6 that the different miscanthus genotypes exhibit extensive variation in both biomass composition and characteristics relevant for energy use and that these are affected by their growing environment and crop management (mainly harvest).

#### Genotypic differences in biomass composition and properties

**Figure 4:**
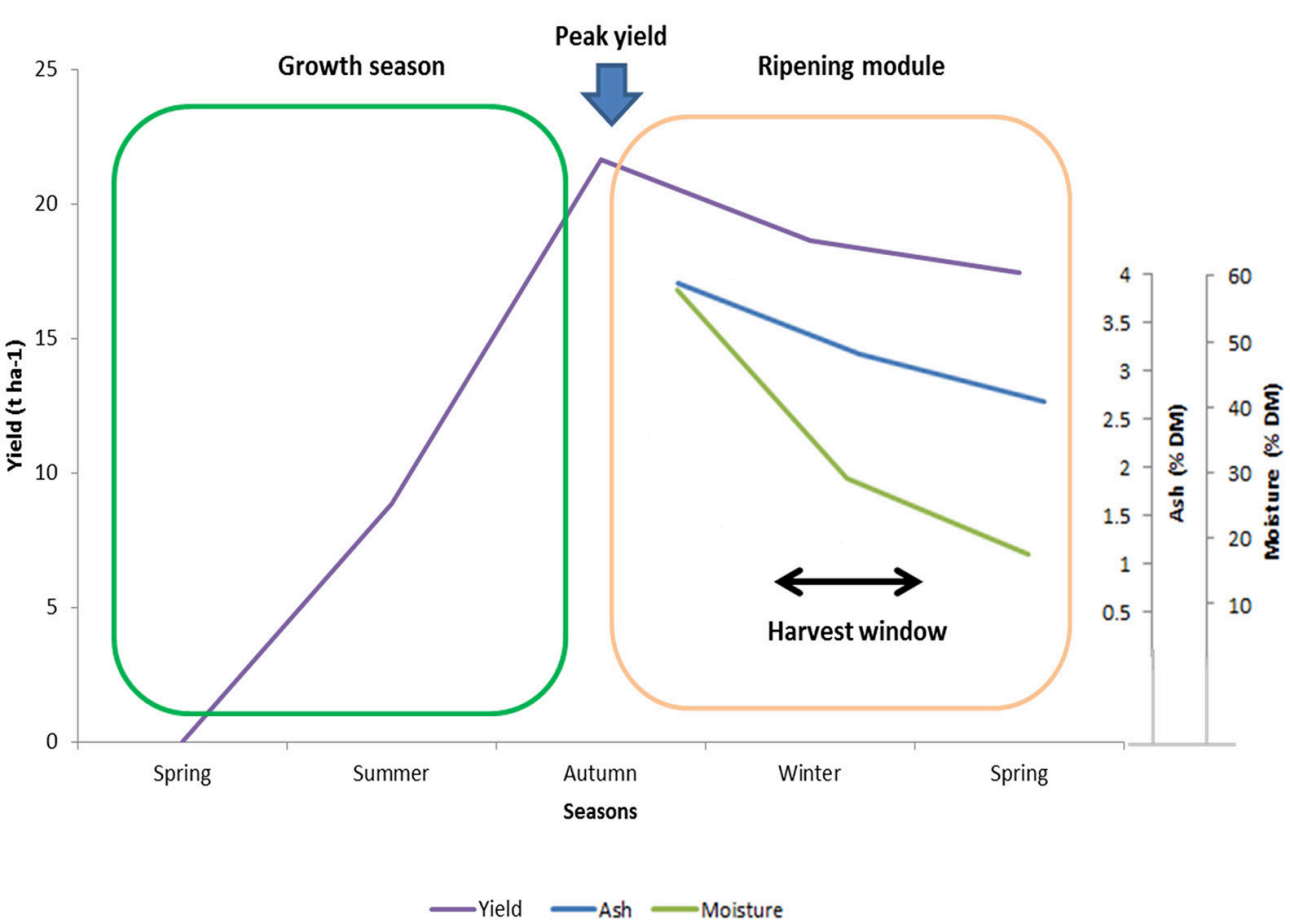
**Average yield accumulation during the growing season and changes in combustion-quality-relevant traits (moisture and ash content) from autumn to spring for the leading clone OPM-06 grown in Stuttgart in the third and fourth year after establishment**.

The moisture content of miscanthus biomass is mainly determined by harvest date (see Figure 4), but is also affected by genotypic variation resulting from morphological differences and senescence patterns. Data from the Blankney Estate large-scale (5-ha) trial in WP5 show that shorter-growing hybrids with thinner stems had lower moisture content (below 13% in the standing crop in 2015), significantly higher bale weight (650 kg vs. 500 kg for *M*. × *giganteus*, with less string breakages) and require about 20% less power for pelleting. However, short-statured types were lower-yielding than *M*. × *giganteus* and the pellets were ~5% less dense.

A trade-off between biomass yield and quality was also observed for the production of biomass for combustion. The concentration of combustion-critical elements declines over winter, as does the biomass yield (Figure 4). Therefore, for combustion purposes, we recommend genotypes with the best combination of good combustion qualities and relatively low biomass losses (and high biomass potential) such as OPM-11 for Adana/Turkey, OPM-03, OPM-06 and OPM-09 for Stuttgart/Germany and OPM-06, and OPM-09 for Moscow/Russia.

Of the eight compositionally diverse *M. sinensis* genotypes evaluated in a field trial in Wageningen, biogas yield ranged from 441 to 520 ml/g dry matter and glucose yield for fermentation ranged from 146 to 208 g/kg dry matter (in very mild processing conditions). Furthermore, variation in genotype performance for these value chains was found to correlate strongly with cell wall compositional characteristics, such as contents of lignin, hemicellulosic polysaccharides, arabinose, *trans*-ferulic acid, *para*-coumaric acid and ratios of these cell wall components. Biogas yield and saccharification efficiency were not highly correlated to each other, although they were both influenced by some of the above compositional characteristics. Nonetheless, some genotypes performed relatively well in both value chains. Unfortunately, these genotypes were not the best-performing genotypes in terms of yield. Thus, one of the challenges for the future is the crossbreeding of biomass-quality and biomass-yield-related traits.

The large variations observed in genotype performance indicate that, by developing and utilizing higher-quality feedstocks, vast improvements could be made in processing efficiency for these value chains.

#### Effect of environmental factors, especially abiotic stress, on cell wall composition

Variation in biomass composition was also shown to be highly influenced by environmental factors. Location accounted for a large part of the variation in cell wall composition in 15 genotypes that were evaluated across six locations in Europe and Russia. Some of this environmental influence can be explained by differences in relative stand maturity during the establishment phase of the trials, but it was still significant after the third growing season. Stand maturity was also found to affect cell wall composition. The cell wall composition in the first growing season had a low predictive value of that in the third growing season. However, cell wall composition in the second year was predictive of that in the third year with reasonable accuracy across all locations. Significant genotype-by-location interaction was seen for cell wall, cellulose, hemicellulose, and lignin contents, indicating that the ranking of genotypes in terms of cell wall components varied across locations. Some genotypes showed considerably more sensitivity to environmental factors than other more stable genotypes. The environmental influence on biomass quality is substantial and should be taken into account when matching genotype, location and end-use of miscanthus.

As lignocellulosic feedstocks are low-value, high-volume commodities, most scenarios consider their cultivation on low-quality/marginal land where the occurrence of various abiotic stresses is highly probable. The fact that agricultural inputs need to be minimized on such land may lead to additional stresses. As miscanthus is seen as a robust perennial crop with high potential for low-quality/marginal soils (Quinn et al., 2015), it is very likely to experience abiotic stresses during its cultivation. Apart from the adverse effects of abiotic stresses on plant growth, another challenge is the fact that abiotic stresses result in changes in cell wall architecture and that in some cases these can lead to a reduction in the industrial quality of the biomass. It has been shown that subjecting plants to abiotic stress treatments often results in cell wall biosynthesis genes being differentially expressed (Moura et al., 2010; Frei, 2013; Le Gall et al., 2015; Tenhaken, 2015). However, there have not been many investigations into the specific effects of the various abiotic stresses on cell wall composition and biomass quality (Tenhaken, 2015).

The OPTIMISC project assessed the effects of the abiotic stresses drought, salinity and cold on miscanthus biomass quality (WP3, WP4, WP6). The abiotic stress treatments were found to lead to substantial changes in biomass composition. Drought stress caused significant reductions in cell wall and cellulose content and a significant increase in hemicellulosic polysaccharides. However, it had only a small effect on lignin content. However, this effect can hardly be separated from the effect of increasing lignin content with maturity of the crop. It was hypothesized that the reduction in cellulose is the result of an increase in osmolyte production at the expense of cellulose as a strategy for maintaining turgor at a lower water potential. Cold stress caused a significant decrease in cell wall, cellulose, and lignin content, again with a significant increase in hemicellulosic polysaccharides. The same trends were observed in response to salt stress, but the effects were smaller.

Overall, the main response observed to all of these abiotic stresses was a decrease in cellulose content and a concomitant increase in hemicellulosic polysaccharides. The reduction in cellulose content has a negative impact on the industrial quality of the biomass for biofuel production, as it implicates a reduction in the main source of fermentable sugars. However, as also seen in the drought-treated samples, the increase in hemicellulosic polysaccharides led to a substantial increase in saccharification efficiency of the biomass. There is often a positive correlation between hemicellulosic polysaccharides and increased cell wall degradability, as an increase in these highly branched polysaccharides is associated with a reduction in crystallinity (Xu et al., 2012). Thus, although the stressed samples contain a lower amount of fermentable sugars, they are more easily extracted. This could potentially reduce processing costs for many potential value chains, including biofuel production. The higher degradability of plants experiencing abiotic stresses makes miscanthus an interesting crop for exploitation of marginal soils for the production of second-generation biofuel.

#### Harvest regime

Several of the OPTIMISC trials (WP4, WP5) included evaluations of the effects of different harvest regimes on miscanthus biomass yields. As Figure 4 shows, the yield reaches a peak in autumn and then decreases, mainly due to leaf loss. The assessments concentrated on the effect of harvest time on biomass yield and quality and investigated whether multiple cutting systems could improve yield performance.

For *M. sinensis*, a double-cut harvest (summer cut in July, winter cut in February) was shown to yield significantly less biomass than a single-cut harvest in February. Averaged over eight genotypes, the double-cut regime yielded an annual biomass of ~2.4 t DM/ha while the single-cut regime yielded ~6.3 t DM/ha for the first complete growing season after establishment. The weather conditions in summer 2015 favored a higher biomass quality for ethanol and biogas value chains, but the yield penalty of an early cut was too substantial to recommend a summer cut for any of the miscanthus value chains considered.

Similar results were observed in a cutting tolerance trial using *M*. × *giganteus* (Kiesel and Lewandowski, 2017). In this trial, a double-cut harvest regime (first green cut in July, second green cut in October) and two single-cut harvest regimes (early harvest in August and late harvest in October) were compared with a conventional spring harvest. The double-cut and the early single-cut harvest regime showed serious yield decline the following year, indicating that both regimes were not tolerated by the crop and are not sustainable in terms of yield formation. The harvest in late October delivered very high and stable yields of 25–28 t DM ha^−1^, suggesting that *M*. × *giganteus* can tolerate a green harvest at this time. Relocation of carbohydrates was identified as an important factor influencing the cutting tolerance. Our hypothesis is that the autumn-harvested crop had enough time to relocate sufficient carbohydrate reserves to the rhizome before the harvest in late October (Kiesel and Lewandowski, 2017).

The biomass quality of green-harvested miscanthus for biogas production and consequently substrate-specific biogas yield declined with later harvest dates. October was identified as the most promising and cutting-tolerant harvest date for biogas production. On average it delivered a 45% higher methane yield than the conventional winter harvest, due to higher biomass yield and improved biomass quality.

However, the early green harvest led to a biomass yield decline the following year due to insufficient cutting tolerance. This lower biomass yield was not compensated by the higher substrate-specific biogas yield. Therefore, cutting tolerance was identified as a crucial factor for the long-term productivity of green-harvested miscanthus.

Cutting tolerance is also relevant for the use of miscanthus biomass for protein and chlorophyll production. These can be extracted from the biomass prior to its processing for bioenergy or other applications. Chlorophyll is used as a food additive, whereas protein is used as a feed additive. As such, both are important added-value bioproducts and can contribute to the value of miscanthus biomass in the biorefinery chain. In the large-scale field trials at Blankney and Stuttgart, it was found that harvesting the stay-green OPM-111 (*M. sinensis*) later than early July resulted in a significant decrease in both chlorophyll and protein content. At harvest earlier than July, the protein content of leaves and stems were about 12 and 11% of DM, respectively. At Blankney, the chlorophyll content reached up to 3.5% of DM in leaves and 2.8% of DM in stems. At Stuttgart however, leaf and stem chlorophyll contents only reached about 2.5 and 1.8% of DM, respectively. We concluded that miscanthus can probably hardly compete with the existing methods for chlorophyll extraction from perennial ryegrass.

#### Technologies for harvesting and logistics

Harvesting miscanthus is a fuel- and labor-intensive process (depending on harvest procedures), and has the largest cost and environmental impact (in terms of fuel usage) for a producer. For this reason, it is important to gather data that can help growers make use of methods best suited to their existing equipment and facilities. In addition, data is required that take the variation in harvest efficiencies of the different genotypes into account to allow farmers to cultivate the genotype best suited to their harvesting needs, thus maximizing profitability in the biomass value chain.

Harvesting techniques, climatic conditions and plant morphology all interact to affect biomass quantity and quality and the resultant options for downstream biomass utilization (see Section Genotypes suitable for processing and use—biomass quality). Self-propelled forage harvesters (normally used for maize) have been successfully used to produce chips from *M*. × *giganteus* in the UK, France and Germany following cold winters, which force the crop to ripen with a moisture content below 25%. This direct chipping approach results in biomass losses of only 5% (Meehan et al., 2013). The chips dry well in covered storage. However, miscanthus chips have a number of drawbacks. Firstly, they have a low bulk density (150 kg m^−3^), which leads to high storage costs and limits the location of markets to within the proximity of the available crop. Secondly, the low bulk density reduces the fuel mass in the combustion chamber, which lowers the thermal output of most boilers. Thirdly, unless the chips have been produced using a high-precision chop forage harvester, bridging, and clogging can be a problem with automated feed systems.

The harvesting experiments at Blankney in WP5 led to the following conclusions:

– Large self-propelled direct chipping harvesters with 7.5-m cutting widths have high throughputs and are potentially more fuel (3%) and time (~10%) efficient than machines with a 4.5-m cutting width.
– Farmers (or machine rings) will most probably harvest with the locally available technology in order to minimize additional capital costs. Therefore, it is likely that smaller harvesting machines will be used. Harvesting speed and efficiencies do not represent a bottleneck to deployment. As the scale of planting increases, the machinery will develop to match demands.
– Moisture content of the different hybrid types harvested at Blankney ranged from 13 to 20% of DM in April 2015. The hybrids with low moisture content are the most amenable to harvest by self-propelled direct chipping harvesters, since no degradation of the biomass occurs during storage at these low moisture levels.
– In mild winters, where senescence is incomplete in non-flowering genotypes such as *M*. × *giganteus*, mowing and then windrowing before baling will remain an important harvest method even though harvest losses are higher.

OPTIMISC also investigated the pelleting of miscanthus biomass. All the pellets produced are described as “good, hard, and durable.” The highest bulk density (810 g/l) was achieved using *M*. × *giganteus* (OPM-09) biomass and the lowest (664 g/l) was observed for OPM-12. The highest percentage of fines (small particles of un-pelleted material) occurred in OPM-52 (25%) and the lowest in OPM-12 (16%).

Large-scale commercial pelleting tests showed that all miscanthus hybrids could be successfully pelleted. Slight adjustments to the machinery normally used for wood pellets are needed with *M*. × *giganteus* to avoid overheating of the press. All the new (softer-stemmed) hybrids tested had lower pressing resistances and therefore lower die temperatures and power requirements.

The different miscanthus hybrids tested showed significant variation in pelletability. As was expected, *M*. × *giganteus*, with its hard, stiff stems, was the most difficult to pellet, but it gave the highest pellet bulk density.

The energy costs of large-scale pellet production can vary from 40 to 80€/t pelleted biomass, at a capacity of approximately 3 t/h. The final cost of production also depends on the wear and tear of pellet press parts (die and rollers), and there is a significant correlation between this wear and tear and biomass composition and structure.

The calorific values of the pellets from the different hybrids varied slightly, but there was wide variation in ash and chloride contents. The biomass of the softer-stemmed hybrids had both a lower moisture content at harvest and also lower levels of ash and chlorine after pelleting than that of *M*. × *giganteus*.

### Miscanthus value chains—options and implementation

In OPTIMISC, the economics as well as GHG- and fossil-fuel-saving potentials of seven miscanthus-based value chains were analyzed in detail in WP7. Table 8 ranks the potential GHG savings by different miscanthus-based value chains for sites in north-eastern Europe (data from the Moscow/Russia site), for Central Europe (data from the Stuttgart/Germany site), and for southern Europe (data from the Adana/Turkey site).

**Table 8:** **Optimized miscanthus-based value chains**.

	**Biomass production genotype**	**Harvest**	**Pre-treatment**	**Processing**	**End product**
**NORTH-EASTERN EUROPE**					
Insulation	OPM-06 (10, 9)	March	Steam explosion	Mixing/pressing	Insulation material
Heat–chips	OPM-06 (10, 9)	March	Chipping	Combustion	Heat
CHP–bales	OPM-06 (10, 9)	March	Baling	Combustion	Heat + Power
CHP–pellets	OPM-06 (10, 9)	March	Pelleting	Combustion	Heat + Power
Heat–pellets	OPM-06 (10, 9)	March	Pelleting	Combustion	Heat
Biogas	OPM-06 (10, 14)	October	Ensiling	Anaerobic digestion	Heat + Power
Ethanol	OPM-06 (10, 9)	March	Thermo-chemical	Fermentation	Ethanol
**CENTRAL EUROPE**					
Insulation	OPM-06 (3, 10)	March	Steam explosion	Mixing/pressing	Insulation material
Heat–chips	OPM-06 (3, 9)	March	Chipping	Combustion	Heat
CHP–bales	OPM-06 (3, 9)	March	Baling	Combustion	Heat + Power
CHP–pellets	OPM-06 (3, 9)	March	Pelleting	Combustion	Heat + Power
Heat–pellets	OPM-06 (3, 9)	March	Pelleting	Combustion	Heat
Ethanol	OPM-06 (3, 10)	March	Thermo-chemical	Fermentation	Ethanol
Biogas	OPM-06 (3, 11)	October	Ensiling	Anaerobic digestion	Heat + Power
**SOUTHERN EUROPE**					
Insulation	OPM-09 (11, 14)	March	Steam explosion	Mixing/pressing	Insulation material
Heat–chips	OPM-09 (11, 14)	March	Chipping	Combustion	Heat
CHP–bales	OPM-09 (11, 14)	March	Baling	Combustion	Heat + Power
CHP–pellets	OPM-09 (11, 14)	March	Pelleting	Combustion	Heat + Power
Heat–pellets	OPM-09 (11, 14)	March	Pelleting	Combustion	Heat
Ethanol	OPM-09 (11, 14)	March	Thermo-chemical	Fermentation	Ethanol
Biogas	OPM-09 (11, 6)	October	Ensiling	Anaerobic digestion	Heat + Power

#### Carbon mitigation and fossil-energy substitution potentials

For all miscanthus energy and material applications, OPM-06 is most suitable in north-eastern and Central Europe, followed by OPM-10 and OPM-09 in north-eastern and OPM-03 and OPM-09 in Central Europe. In southern Europe, OPM-09 (*M*. × *giganteus*) proved most suitable for all the miscanthus-based value chains analyzed, followed by OPM-11 and OPM-14 or OPM-06 for biogas. This means that *M*. × *giganteus* proved a feasible choice for all locations and applications. The suitability of the genotypes was determined according to yield and quality performance with regard to anticipated use.

The optimal harvest time differs for each value chain. For combustion, a late harvest leads to low moisture content and other favorable biomass quality criteria, but also to biomass yield losses. For ethanol and biogas production, a green harvest in autumn is optimal (Table 8). For biogas production, high DM yield and low lignin content are important determinants for high biogas yield and can best be achieved by a green cut. A green cut is also a prerequisite for biomass ensilage.

The highest biomass yields as well as the highest GHG- and fossil-energy savings potentials (up to 30.6 t CO_2eq_/ha^*^a and 429 GJ/ha^*^a, respectively) can be achieved on non-marginal sites in Central Europe. On marginal sites limited by cold (Moscow/Russia) or drought (Adana/Turkey) savings of up to 19.2 t CO_2eq_/ha^*^a and 273 GJ/ha^*^a (Moscow) and 24.0 t CO_2eq_/ha^*^a and 338 GJ/ha^*^a (Adana) can be achieved.

The GHG and fossil-energy savings are highest where miscanthus biomass is used as construction material (our analysis uses the example of insulation material). A high GHG- and fossil-energy-saving potential was also found for domestic heating on account of the short transportation distance. Pelleting is only advantageous in terms of the minimization of GHG emissions and energy consumption where biomass is transported over a long distance, for example for heat and power production in CHP. Pelleting requires additional energy, but at the same time reduces the energy required for transport due to its higher density.

The lowest GHG- and fossil-energy-saving potentials were found for power production via the biogas pathway, followed by bioethanol. However, this result is strongly influenced by the assumptions that (a) only 50% of the available heat is used and (b) transport distance from the field to the biogas plant is relatively long (15 km). A biogas chain with 100% heat utilization and lower transportation distances would perform better. It can be concluded that for power generation from miscanthus biomass, the most favorable pathway is combustion for base load power, and biogas to cover peak loads.

The economics of biomass production for different value chains are shown in Table 9 for the example of the Stuttgart site (Germany).

**Table 9:** **State-of-the-art biomass supply costs, allocated costs (assessed as difference between biobased and fossil resources), and carbon mitigation costs of each value chain at the Stuttgart site**.

**Value chain**	**Biomass supply costs**		**Allocated costs**		**CO_2_ mitigation costs [€ (t CO_2eq_)^−1^]**
1) Small-scale combustion: chips	0.46	€ct (MJ_th._)^−1^	–0.77	€ct (MJ_th._)^−1^	–78.33
2) Small-scale combustion: pellets	0.79	€ct (MJ_th._)^−1^	–0.43	€ct (MJ_th._)^−1^	–49.65
3) Large-scale combustion: bales	6.25	€ct (MJ_el._)^−1^	5.6	€ct (MJ_el._)^−1^	82.52
4) Large-scale combustion: pellets	6.15	€ct (MJ_el._)^−1^	5.5	€ct (MJ_el._)^−1^	83.54
5) Large-scale bioethanol production	14.80	€ct (MJ_Bioethanol_)^−1^	11.52	€ct (MJ_Bioethanol_)^−1^	1737.56
6) Medium-scale biogas production	2.15	€ct (MJ_el._)^−1^	1.47	€ct (MJ_el._)^−1^	93.69
7) Large-scale insulation plant	27.69	€ (m^3^)^−1^	28.53	€ (m^3^)^−1^	70.75

Biomass supply costs are assessed here as the costs of producing, densifying, and transporting the biomass from the farm to the unit where the biomass is burned or processed into ethanol or insulation material. They range from 78€ per ton dry mass of chips (for local, small-scale production) and 79€ per ton silage (50% water) for biogas production up to about 140€ per ton dry mass of bales for the production of insulation material, ethanol, and pellets.

In a comparison with the production of energy from fossil fuels, small-scale combustion of chips proved to be highly profitable. The pelleting of biomass increases the cost by about 30%, but the cost per KWh thermal energy produced still remains comparatively low. Both options lead to negative carbon mitigation costs (Table 9).

When electricity is produced in a medium-scale 5 MW CHP power plant, carbon mitigation costs are about 83€ per ton avoided CO_2equivalents_ for biomass supply as bales or pellets, assuming a transport distance of 400 km (Table 9). To make CHP electricity a viable option for electricity production from miscanthus biomass, transportation costs need to be reduced. For bioethanol, costs of about 24€ ct per liter stem from biomass supply. Here too, reduction of transport distances is an important factor in lowering biomass supply costs. For insulation material, biomass supply costs per m^3^ are of the order of 28€, if a transport distance of 400 km is assumed. This can compete with the market price of glass wool. The competitiveness of miscanthus insulation can be improved by its cultivation closer to the insulation material production site.

There was a clear effect of yield level on the cost per unit of biomass. For conditions comparable to those prevailing at the Stuttgart site, the cost of bale harvest was 28.9€/t DM for a yield of 15 t/ha. This decreased to 23.5€/t DM when a higher yield of 18 t DM/ha was assumed. This example reveals the limitations of miscanthus cultivation on marginal land, where costs per unit produced are higher and not always compensated for by lower costs for lease of land. However, the results of the OPTIMISC project should lead to an increase in biomass yield of miscanthus cultivated on marginal land, as novel genotypes outyielded *M*. × *giganteus* at three of the five experimental sites. At the Stuttgart site, OPM-06 had a 20% higher yield than *M*. × *giganteus*. New hybrids from the gene pools tested and characterized in OPTIMSC are expected to become commercially available in the near future.

Another option for alleviating the problem of marginal yield levels is cultivation on larger-sized plots. Growing miscanthus for combustion on a 20-ha plot instead of a 2-ha plot can decrease biomass costs by 18% (KTBL, 2012). As cultivation on marginal land involves lower opportunity costs than on high-yielding farmland, lower economic returns are acceptable. When grown on fields where annual crops often fail, the perennial crop will always give some return and thus can be more attractive, even with moderate yield levels. In Iowa/USA, it is estimated that there are good opportunities for miscanthus cultivation on 10–20% of marginal corn land, where farmers lose money every year (Heaton, 2014).

The OPTIMISC project also created new perspectives and opportunities through the option of higher prices for miscanthus with higher added value for industry. Research on quality aspects of different genotypes for specific end uses allowed the identification of novel genotypes which can incorporate improved quality characteristics at field level. For example, it was shown that there is scope for development of new varieties with considerable potential to reduce pre-treatment costs for bioethanol production. Bioethanol yield after mild treatment of lignocellulosic biomass is a good indicator of possible savings in industrial production. At Stuttgart/Germany, hybrid OPM-06 had a 37% higher ethanol yield than *M*. × *giganteus* (OPM-09) after mild treatment. This should lead to higher biomass prices due to cost reduction in industrial processes.

## Outlook: how to support the implementation of miscanthus production and use in Europe

The two most important areas where technological advances can be made are breeding programs and development of agricultural equipment for miscanthus production. Other factors are access to markets and the development of a robust supply chain from the farmer to the end-user. The development of high-value biomass applications, such as biochemicals and biocomposites, should also be encouraged. A stronger engagement of farmers in the value chain through “on-farm biorefining” concepts would increase their income opportunities, allowing them to market high-value products instead of low-value biomass. It is also recommended that the ecological potential of miscanthus should be acknowledged in the European Common Agricultural Policy (CAP), for example by giving further consideration to the development of so-called “greening measures.” These recommendations are elaborated below:

### Ecological benefits of perennial biomass crops need to be recognized

Remuneration for non-market ecosystem services should include funding for particularly high-service provision, e.g., flood risk reduction, soil protection, nitrate mitigation etc.

### Replace less sustainable biomass with perennial biomass crops (PBC)

About six million ha of agricultural land in the EU are used for so-called “first generation” energy and industry crops. Rapeseed and maize are the most prominent examples. The replacement of these intensively managed annual crops by perennial biomass crops could be a priority for reducing nitrate leaching, erosion and the use agrochemical use, and increasing soil carbon sequestration and biodiversity. Miscanthus could replace maize for biogas production, if fermentation techniques are adapted or the biomass pre-treated.

### Support the development of miscanthus varieties adapted to marginal land

Miscanthus shows good potential to make use of land, which is marginal or difficult to manage, or for land restoration. However, marginal production conditions can also result in low profit margins (van Dam et al., 2005) which are not compensated for by lower land costs. Therefore, crop management systems that ensure safe establishment and optimal management in these conditions need to be developed and stress-tolerant genotypes are required.

Plant genetic improvement, even of major agricultural crops such as wheat, is subject to significant market failure (Moran et al., 2007). This leads to underinvestment in breeding on the private market, as the incentive is insufficient for the level of investment optimal for society. This failure is likely to be even greater for perennial biomass crops, particularly for adaptation to marginal land. The OPTIMISC project identified the tolerance of miscanthus to the abiotic stresses that characterize marginal land. Insights were gained into the available land resources and thus the potential market for improved planting material. These findings lead us to suggest a miscanthus strategy that includes a plan for appropriate public investment in plant breeding, also through partnerships with private-sector breeders. This should stimulate demand for “upstream” research, an area that also requires long-term support.

### Technical barriers to the implementation of PBC on marginal land need to be overcome

The estimated area of land under miscanthus cultivation in the EU is currently about 20,000 ha and is decreasing in many regions. Insufficient development of cultivars and production technology, along with high costs for agricultural inputs, land and labor, result in high production costs for a relatively low-value biomass. Although they are amortized over a cultivation period of 4 to 25 years, establishment costs for miscanthus are high and need to be reduced.

The development of agricultural machinery, such as planting and harvesting equipment, will remain insufficient unless it finds a larger market. Today, farmers often use self-made equipment, such as adapting potato harvesting machines for the harvest of miscanthus rhizomes. In addition, the development of service units, e.g., machinery cooperatives, will only develop once miscanthus production reaches a significant scale.

Farmers hesitate to grow miscanthus because it involves dedicating their land to long-term biomass production. They will only be willing to do this if biomass markets are reliable or if long-term contracts are available in recognized supply chains. Therefore, the development of biomass marketing structures should be supported by agricultural policies.

The main current application of miscanthus biomass is for bulk heat and power production—a comparatively low-value market whose value depends on the price of fossil fuels used in large-scale heat and for electricity generation. Complementary to these existing markets, there is a need for programs that support smaller-scale but higher-value applications of miscanthus biomass to develop new, attractive market options. These should include options for “on-farm biorefineries” that help keep a higher proportion of the value generated from biomass on the farm. The development of on-farm biorefinery concepts, which allow decentralized biomass densification and valorization, can help involve farmers in local biobased value chains.

## Author contributions

The authors contributed in the following roles: IL University of Hohenheim, as corresponding author and coordinator of the project. JC as Work Package Leader. LT as Work Package Leader. GV as Work Package Leader. KS as Work Package Leader. KM as Work Package Leader. OK as Project Manager. AA, CC, OD, ID, KF, SF, GH, AH, LH, YI, NK, AK, PL, HeM, MM, HiM, CN, MÖ, IR, HS, IT, TV, MW, and QX as Project Coworkers.

### Conflict of interest statement

The authors declare that the research was conducted in the absence of any commercial or financial relationships that could be construed as a potential conflict of interest.
